# Supplementary material for: The ‘social gradient' in primary liver cancer in France: A national observational study
Source: JHEP Rep. 2025 Sep 5;7(11):101585. doi: 10.1016/j.jhepr.2025.101585 (PMC12519276; doi:10.1016/j.jhepr.2025.101585)
Supplement: Multimedia component 1 [file mmc1.pdf]

The 'social gradient' in primary liver cancer in France:

A national observational study

Marie Strigalev, David Fuks, Sandrine Katsahian, Lucia Parlati, Ugo Marchese, Maria Conticchio, Charlotte Ronde-Roupie, Alexandra Nassar, Alix Dhote, Vincent Mallet, Stylianos Tzedakis

Table of contents

Table S1…………………………………………………………………………………2

Table S2…………………………………………………………………………………6

Table S3…………………………………………………………………………...……11

Table S4……………………………………………………………………………….. 14

Fig. S1………………………………………………………………….……….....16

Fig. S2………………………………………………………………….……….....17

Fig. S3………………………………………………………………….……….....18

Fig. S4………………………………………………………………….……….....19

**Table S1:** Code dictionary	
**Variable**	ICD-10 and CCAM medical procedure codes
**Acute liver failure**	K720, K729
**Alcohol use disorders**	E244, E511, F101, F102, F103, F104, F105, F106, F107, F108, F109, F1020, F1021, F1022, F1023, G312, G621, G721, I426, K292, K70, K852, K860, O354, Z502, Z714, Z721
**Alcoholic liver disease**	K70
**Biliary duct surgery**	HMFA010, HMFA009, HMLA001, HMLC001, HMFA001, HMFA002, HMFA005, HMFA006, HMFC003, HMFC005
**Chronic kidney disease**	E102, E112, E122, E132, E142, I151, JAHB001, JAHH002, JAHC001, JAHA001, JAHJ006, JAHJ007, N02, N04, N06, N07, N08, N181, N182, N19, N25, N083
**Chemotherapy for Cancer**	Z511, Z512
**Chemoembolization**	EDLF017, EDLF016, EDLF015, EDLF014
**Cirrhosis**	I859, I864, I982, I9829, K703, K717, K743, K744, K745, K746, K766
**Decompensated Cirrhosis**	R17, K704, K711, K72, K767, I280, R18, J948, K65, HPHB003, HPJB001, EHCA003, EHCA006, EHCA009,

	EHCA007, EHCA004, EHCA002, EHCA005, EHCA010, EHCA001, HEPA005, HEPA004, HEPA007
**Congestive heart failure**	I099, I110, I130, I132, I255, I420, I425, I426, I427, I428, I429, I43, I50, P290
**Chronic Obstructive Pulmonary Disease**	I278, I279, J40, J41, J42, J43, J44, J45, J46, J47, J60, J61, J62, J63, J64, J65, J66, J67, J684, J701, J703
**Diabetes mellitus complicated**	E102, E103, E104, E105, E107, E112, E113, E114, E115, E117, E122, E123, E124, E125, E127, E132, E133, E134, E135, E137, E142, E143, E144, E145, E147, H360, N083, H280, G632
**Diabetes mellitus uncomplicated**	E100, E101, E106, E108, E109, E110, E111, E116, E118, E119, E120, E121, E126, E128, E129, E130, E131, E136, E138, E139, E140, E141, E146, E148, E149
**Gastrectomy**	HFPA001, HFFA009, HFFA002, HFFA003, HFFA008, HFFA006, HFFA005, HFMA005, HFFC001, HFFC002, HFFC012, HFFC017
**Gastro Esophageal Varices Bleeding**	I850, I983, I9820, EHBD001, EHNE002, HESE001, HESE002
**Hepatitis B, C and D virus**	B181, B162, B169, B182, B160, B161, B180, B170

**Hepatocellular carcinoma**	C220
**Hepatopulmonary and Hepatorenal Syndrome**	I280, K767
**Hepatic Encephalopathy**	K704, K711, K74
**Intrahepatic cholangiocarcinoma**	C221
**Jaundice**	R17
**Liver biopsy**	HLHB001, HLHJ003, HLHJ006, HLHH006, HLHJ005, HLHH007, HLHH001, HLHH005
**Liver resection**	HLFA003, HLFA009, HLFA011, HLFA020, HLFA006, HLFC002, HLFC004, HLFC027, HLFC032, HLFA004, HLFA005, HLFA007, HLFA010, HLFA017, HLFA018, HLFC037, HLFC801
**Liver ablation (radiofrequency, microwave)**	HLNN900, HLNK001, HLNM001, HLNA007, HLNC003
**Liver failure**	K720, K729, K711, K763
**Liver Transplantation**	Z944, HLEA001, HGEA002, HLEA002, HGEA004

**Metastasis intra- and extra-hepatic**	C787, C780, C782, C783, C781, C784, C788, C798, C799, C793, C797, C786, C482, K669, K668, C796, C795
**Non Viral Non Metabolic Chronic Liver Disease**	E831, E84, FEJF003, FEJF006, FEJF008, E830, N163, I820, K743, K754, Q44, T864, Z944
**Obesity**	E66
**Portal Vein Thrombosis**	I81
**Portal Hypertension**	K766
**Phlebitis and Thrombophlebitis**	I80
**Palliative Care**	Z515
**Radioembolization**	EDLL001, EDLL002
**Smoking**	F17, Z716, Z720, T652

Table S2: Univariable and multivariable associations with curative treatment of PLC in France 2017 - 2021

**Characteristic**	**Univariable Associations**				**Multivariable Associations**	
	**Overall, N = 62,351**[1]	**Palliative treatment or palliative care, n = 45,657 (73%)**[1]	**Curative treatment, n = 16,694 (27%)**[1]	**p-value**[2]	**aOR (95% CI)**	**p-value**
**Deprivation**	27,872 (44.7%)	20,916 (45.8%)	6,956 (41.7%)	**<0.001**	0.89 (0.85, 0.92)	**<0.001**
**Advanced PLC**	33,317 (53.4%)	24,485 (53.6%)	8,832 (52.9%)	0.11	0.58 (0.55, 0.62)	**<0.001**
**Late-stage disease**	8,188 (13.1%)	7,856 (17.2%)	332 (2.0%)	**<0.001**	0.11 (0.10, 0.12)	**<0.001**
**Age, years**	71 (63.0, 78)	72 (64.0, 80)	68 (61.0, 74)	**<0.001**	0.97 (0.97,0.97)	**<0.001**

**Male Sex**	44,138 (70.8%)	31,445 (68.9%)	12,693 (76.0%)	**<0.001**	1.03 (0.99, 1.08)	0.2
**Obesity**	15,371 (24.7%)	10,348 (22.7%)	5,023 (30.1%)	**<0.001**	1.39 (1.31, 1.47)	**<0.001**
**Metabolic syndrome**	8,068 (12.9%)	5,394 (11.8%)	2,674 (16.0%)	**<0.001**	0.96 (0.88, 1.04)	0.3
**Smoking habits**	11,683 (18.7%)	7,768 (17.0%)	3,915 (23.5%)	**<0.001**	1.13 (1.08, 1.19)	**<0.001**
**Alcohol use disorders**	23,285 (37.3%)	15,674 (34.3%)	7,611 (45.6%)	**<0.001**	1.02 (0.97, 1.08)	0.4
**Ischemic heart disease**	7,380 (11.8%)	5,350 (11.7%)	2,030 (12.2%)	0.13	1.02 (0.96, 1.09)	0.5
**Chronic kidney disease**	4,495 (7.2%)	3,080 (6.7%)	1,415 (8.5%)	**<0.001**	1.10 (1.02, 1.19)	**<0.001**
**Chronic obstructive pulmonary disease**	7,679 (12.3%)	5,641 (12.4%)	2,038 (12.2%)	0.6	0.97 (0.91, 1.03)	0.4

**Diabetes**	23,438 (37.6%)	16,540 (36.2%)	6,898 (41.3%)	**<0.001**	1.17 (1.12, 1.23)	**<0.001**
**Viral hepatitis**	6,195 (9.9%)	3,649 (8.0%)	2,546 (15.3%)	**<0.001**	1.40 (1.31, 1.50)	**<0.001**
**Non viral non metabolic chronic liver disease**	3,652 (5.9%)	1,396 (3.1%)	2,256 (13.5%)	**<0.001**	3.84 (3.57, 4.14)	**<0.001**
**Cirrhosis**	21,184 (34.0%)	14,117 (30.9%)	7,067 (42.3%)	**<0.001**	1.42 (1.33, 1.51)	**<0.001**
**CCI**				**<0.001**		
Mild	15,103 (24.2%)	10,239 (22.4%)	4,864 (29.1%)		1 (Ref)	
Moderate	19,283 (30.9%)	13,965 (30.6%)	5,318 (31.9%)		0.75 (0.71, 0.80)	**<0.001**
Severe	27,965 (44.9%)	21,453 (47.0%)	6,512 (39.0%)		0.63 (0.59, 0.67)	**<0.001**

**Liver cancer histology**				**0.002**		
ICC	20,891 (33.5%)	17,582 (38.5%)	3,309 (19.8%)		Ref	
HCC	41,460 (66.5%)	28,075 (61.5%)	13,385 (80.2%)		1.86 (1.76, 1.95)	**<0.001**
**Type of hospital center at diagnosis**				**<0.001**		
Non-referral	35,002 (56.1%)	28,927 (63.4%)	6,075 (36.4%)		Ref	
Referral	27,349 (43.9%)	16,730 (36.6%)	10,619 (63.6%)		2.69 (2.57, 2.80)	**<0.001**
[1]Median (IQR); n (%)						
[2]Wilcoxon rank sum test; Fisher's Exact Test for Count Data						

Adjusted outcome was calculated using a fully adjusted mixed effects generalized linear models. Advanced PLC was PLC with any of decompensated cirrhosis, obstructive jaundice, ascites, portal vein thrombosis, hepatic encephalopathy or portal hypertension bleeding. Late-stage cancer disease was defined as PLC with hepatic or extrahepatic metastasis at diagnosis. The Charlson comorbidity index is a weighted index to predict one-year mortality in patients with multiple comorbidities and was classified in three percentiles higher scores indicating higher frailty and ranging from mild (2-6) to moderate (6-8) and severe (>8), without entering age and liver disease. The ecological deprivation index used in the study was the French deprivation index *Fdep* standardized in quintiles (Q) and deprivation was defined as Q4-Q5. Abbreviations: CCI: Charlson comorbidity index; HCC: hepatocellular carcinoma; ICC: intrahepatic cholangiocarcinoma; PLC: Primary Liver Cancer; aOR = adjusted Odds Ratio; CI = Confidence Interval

Table S3: Association of deprivation and treatment access for PLC patients in France 2017 - 2021

Characteristic	Curative treatment (vs palliative care)		Palliative treatment (vs palliative care)		Curative treatment (vs palliative treatment)	
	**aOR (95% CI)[1]**	**p-value[2]**	**aOR (95% CI) [1]**	**p-value[2]**	**aOR (95% CI) [1]**	**p-value[2]**
**Deprivation**	0.87 (0.83, 0.92)	**<0.001**	0.91 (0.87, 0.95)	**<0.001**	0.93 (0.88, 0.98)	**0.004**
**Advanced PLC**	0.52 (0.48, 0.55)	**<0.001**	0.92 (0.86, 0.97)	**0.003**	0.62 (0.58, 0.66)	**<0.001**
**Late-stage disease**	0.09 (0.08, 0.11)	**<0.001**	0.74 (0.70, 0.78)	**<0.001**	0.15 (0.13, 0.17)	**<0.001**
**Age, years**	0.63 (0.60, 0.67)	**<0.001**	0.67 (0.64, 0.70)	**<0.001**	0.83 (0.79, 0.88)	**<0.001**
**Male Sex**	1.05 (0.99, 1.11)	0.081	1.05 (1.00, 1.10)	0.062	1.00 (0.94, 1.05)	0.9
**Obesity**	1.50 (1.39, 1.61)	**<0.001**	1.25 (1.17, 1.33)	**<0.001**	1.19 (1.11, 1.28)	**<0.001**
**Metabolic syndrome**	0.93 (0.84, 1.03)	0.14	0.98 (0.89, 1.07)	0.6	0.97 (0.88, 1.07)	0.5
**Smoking habits**	1.17 (1.10, 1.25)	**<0.001**	1.16 (1.10, 1.23)	**<0.001**	1.03 (0.97, 1.09)	0.4

**Alcohol use disorders**	1.06 (0.99, 1.12)	0.082	1.04 (0.98, 1.10)	0.2	0.97 (0.92, 1.03)	0.4
**Ischemic heart disease**	1.06 (0.99, 1.15)	0.10	1.08 (1.01, 1.15)	**0.030**	0.98 (0.91, 1.05)	0.5
**Chronic kidney disease**	1.07 (0.98, 1.17)	0.15	0.98 (0.90, 1.06)	0.6	1.10 (1.00, 1.21)	**0.042**
**Chronic obstructive pulmonary disease**	0.99 (0.92, 1.06)	0.7	1.01 (0.95, 1.08)	0.7	0.96 (0.89, 1.03)	0.3
**Diabetes**	1.35 (1.27, 1.43)	**<0.001**	1.32 (1.25, 1.39)	**<0.001**	1.00 (0.94, 1.06)	>0.9
**Viral hepatitis**	1.35 (1.25, 1.46)	**<0.001**	0.89 (0.82, 0.96)	**0.003**	1.42 (1.32, 1.53)	**<0.001**
**Non viral non metabolic chronic liver disease**	3.53 (3.20, 3.89)	**<0.001**	1.12 (1.00, 1.26)	0.061	3.35 (3.04, 3.70)	**<0.001**
**Cirrhosis**	1.31 (1.22, 1.41)	**<0.001**	0.83 (0.77, 0.88)	**<0.001**	1.53 (1.43, 1.64)	**<0.001**
**CCI**						
Mild	Ref		Ref		Ref	
Moderate	0.46 (0.43, 0.50)	**<0.001**	0.39 (0.37, 0.42)	**<0.001**	1.11 (1.04, 1.18)	**0.001**

Severe	0.34 (0.32, 0.37)	**<0.001**	0.28 (0.26, 0.30)	**<0.001**	1.08 (1.00, 1.17)	**0.042**
**Liver cancer histology**						
ICC	Ref		Ref		Ref	
HCC	1.79 (1.68, 1.90)	**<0.001**	0.78 (0.75, 0.82)	**<0.001**	1.93 (1.82, 2.05)	**<0.001**
**Type of hospital center at diagnosis**						
Non-referral	Ref		Ref		Ref	
Referral	4.40 (4.19, 4.63)	**<0.001**	2.63 (2.51, 2.75)	**<0.001**	1.53 (1.46, 1.61)	**<0.001**

[1]Adjusted outcome was calculated using a random-effects generalized linear model adjusted for age, sex, general and liver-related comorbidities, CCI, advanced PLC stage, late-stage disease, tumor histology, type of hospital at diagnosis (referral or non-referral) and deprivation.

[2]p-values were adjusted with the false discovery rate method due to multiple comparisons

Abbreviations: CCI: Charlson comorbidity index; PLC: Primary Liver Cancer; aOR = adjusted Odds Ratio; CI = Confidence Interval

Table S4. General and liver-related characteristics balancing after propensity score matching for patients with primary liver cancer by deprivation group between 2017 – 2021 in France

Characteristic	No Deprivation, n = 27,872[1]	Deprivation, n = 27,872[1]	Difference (95% CI)[2]	P value[3]
**General risk factors**				
**Age, years**	71 (63, 78)	71 (63, 78)	0.00 (-0.01, 0.02)	0.202
**Age, > 70 years-old**	14,166 (51%)	14,021 (50%)	0.01 (-0.01, 0.03)	0.219
**Male Sex**	19,940 (72%)	19,900 (71%)	0.00 (-0.01, 0.02)	0.708
**Obesity**	7,398 (27%)	7,460 (27%)	-0.01 (-0.02, 0.01)	0.553
**Metabolic syndrome**	3,895 (14%)	3,970 (14%)	-0.01 (-0.02, 0.01)	0.361
**Alcohol use disorders**	10,952 (39%)	11,006 (39%)	0.00 (-0.02, 0.01)	0.640
**Past or current smoker**	5,347 (19%)	5,320 (19%)	0.00 (-0.01, 0.02)	0.771
**Ischemic heart disease**	3,323 (12%)	3,365 (12%)	0.00 (-0.02, 0.01)	0.584
**Chronic kidney disease**	2,031 (7.3%)	2,026 (7.3%)	0.00 (-0.02, 0.02)	0.935
**Chronic obstructive pulmonary disease**	3,487 (13%)	3,584 (13%)	-0.01 (-0.03, 0.01)	0.217
**Diabetes mellitus**	10,885 (39%)	10,866 (39%)	0.00 (-0.02, 0.02)	0.869
**CCI**	158 (50)		0.01 (-0.01, 0.03)	0.609
Mild	6,298 (23%)	6,396 (23%)		
Moderate	8,660 (31%)	8,610 (31%)	0.12 (0.08, 0.20)	0.4

Severe	12,914 (46%)	12,866 (46%)		
**Liver-related risk factors**				
Chronic Viral hepatitis	2,401 (8.6%)	2,407 (8.6%)	0.00 (-0.02, 0.02)	0.928
Non viral, non metabolic, chronic liver disease	1,627 (5.8%)	1,626 (5.8%)	0.00 (-0.02, 0.02)	0.986
Decompensated cirrhosis	9,138 (33%)	9,088 (33%)	0.00 (-0.01, 0.02)	0.652
Advanced PLC	15,318 (55%)	15,185 (54%)	0.01 (-0.01, 0.03)	0.258
Late-stage disease	3,545 (13%)	3,598 (13%)	-0.01 (-0.02, 0.01)	0.502
Liver cancer histology			0.00 (-0.02, 0.02)	0.993
HCC	18,715 (67%)	18,716 (67%)		
ICC	9,157 (33%)	9,156 (33%)		

[1]Median (IQR); n (%); [2]Standardized Mean Difference; [3]McNemar's test and paired t-tests for paired data

Standardized mean differences (SMDs) were then used to assess covariate balance, with an SMD ≤ 0.01 indicating negligible differences, values from 0.0101 to 0.030 indicating very small differences, values from 0.0301 to 0.050 indicating small differences, and values > 0.050 indicating substantial differences.

Abbreviations: CCI: Charlson Comorbidity Index; HCC: Hepatocellular Carcinoma; ICC: Intrahepatic Cholangiocarcinoma; PLC: Primary Liver Cancer; CI: confidence interval

Fig. S1: Patient flowchart

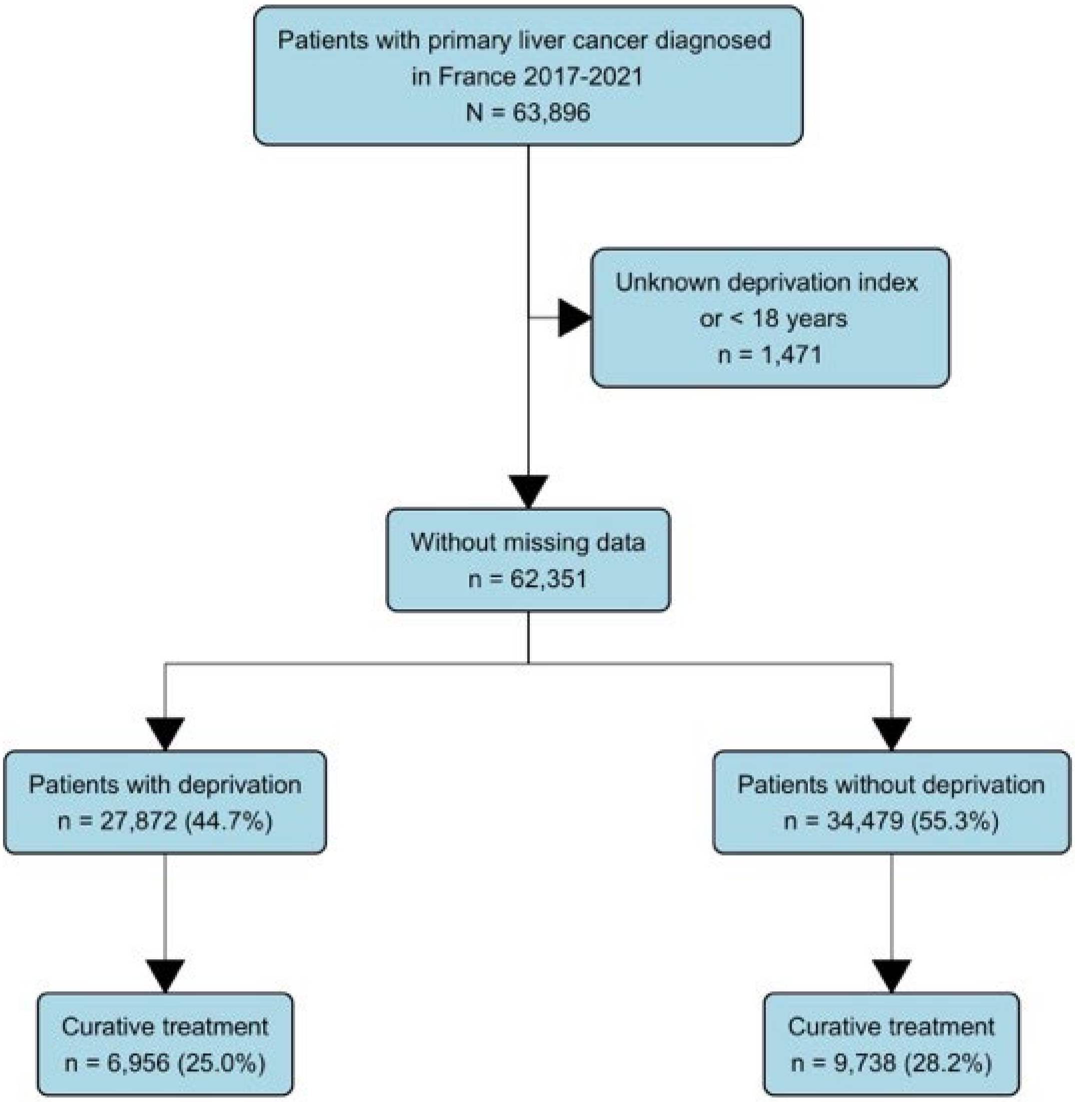

Fig. S2: Interactive map of the departmental variations of social deprivation and curative treatment access among patients with primary liver cancer in France January 1, 2017 - December 31, 2021

Variations of the 5-year incidence of social deprivation (A) and curative treatment access (B) among patients with primary liver cancer (PLC) along the 94 French regional departments between January 1, 2017 and December 31, 2021. Black dots represent expert hospitals in PLC management and dot diameter represents the number of PLC hospitalizations per hospital.

Available at: https://data.mendeley.com/preview/szbg4mnmxn?a=a5f6722b-f9f6-477a-a796-c5aed24f27df

Fig. S3: Correlation between 5-year incidence of deprivation and access to curative treatment in each of the 94 French regional departments.

Footnote: Each dot represents one French department

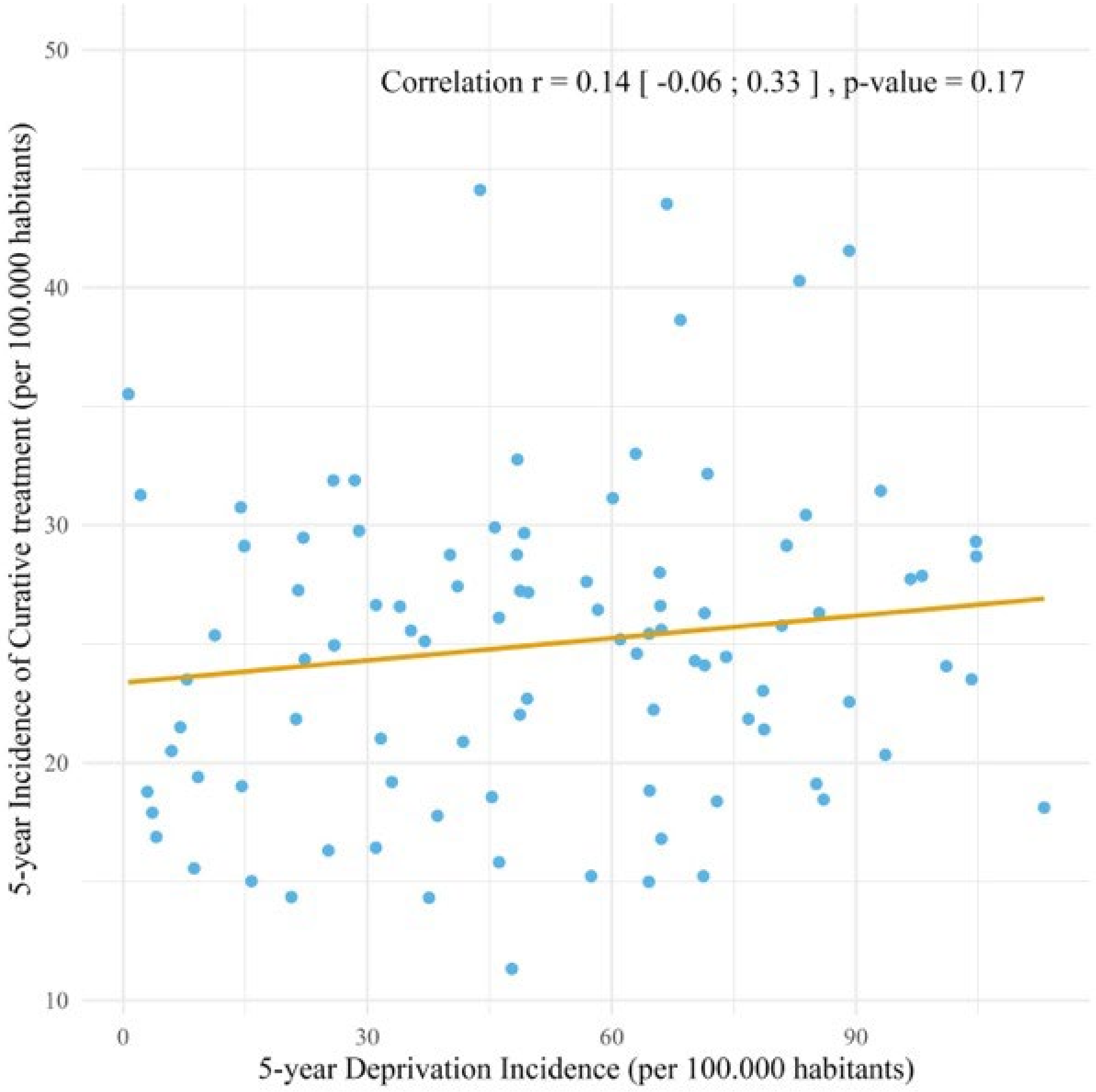

Fig. S4: Plot of confounding covariates before (red line) and after (blue line) balancing on a propensity score.

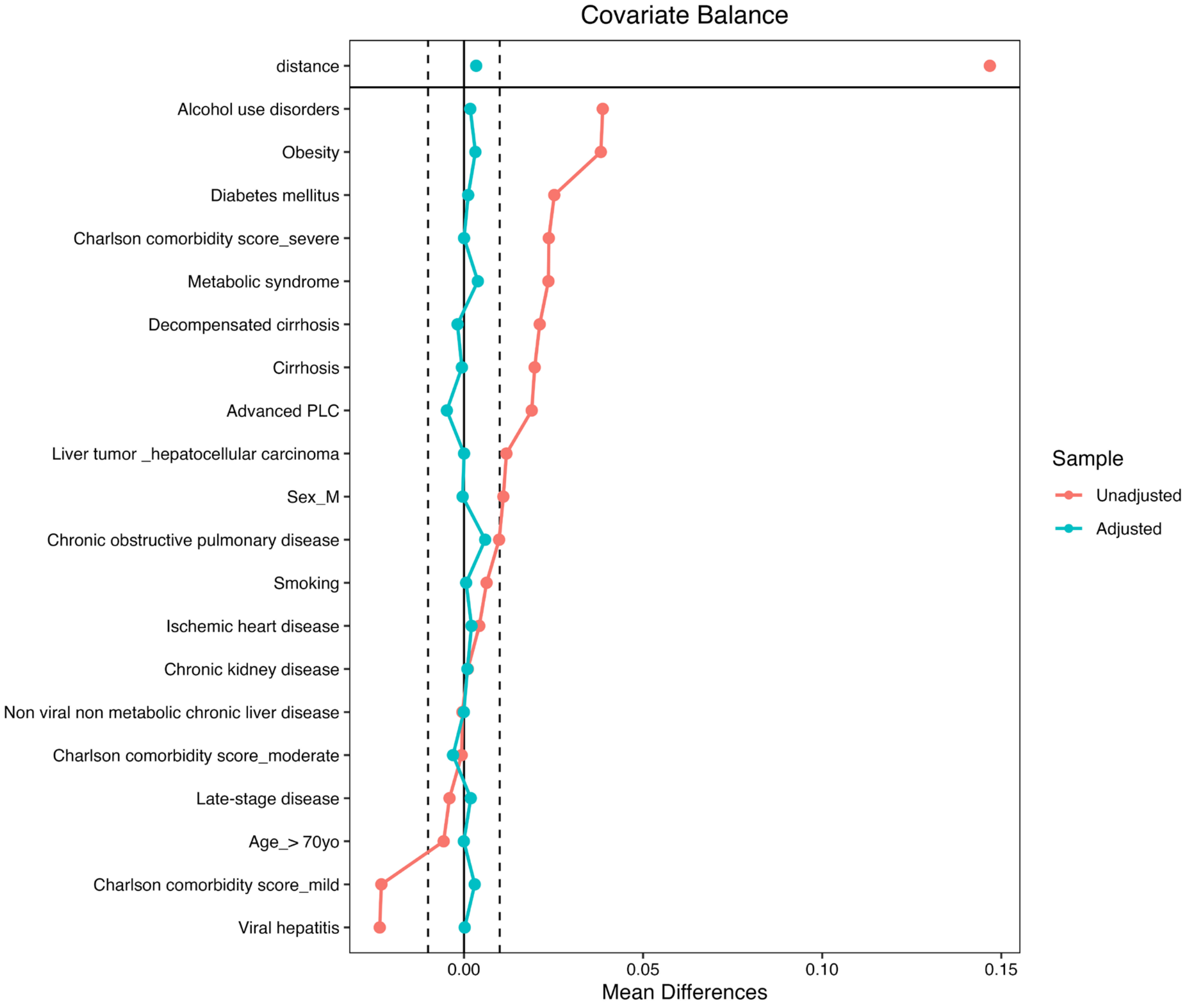

Footnote: Unbalanced general and liver-related comorbidities were included in the propensity score estimation using 1:1 nearest neighbor matching, caliper adjusted at 0.03 without replacement. Standardized mean differences (SMDs) were then used to assess covariate balance, with an SMD $\leq$ 0.01 (dotted line) indicating negligible differences, values from 0.0101 to 0.030 indicating very small differences, values from 0.0301 to 0.050 indicating small differences, and values > 0.050 indicating substantial differences.
