# Supplementary material for: The ‘social gradient' in primary liver cancer in France: A national observational study
Source: JHEP Rep. 2025 Sep 5;7(11):101585. doi: 10.1016/j.jhepr.2025.101585 (PMC12519276; doi:10.1016/j.jhepr.2025.101585)
Supplement: Multimedia component 4 [file mmc4.pdf]

JHEP|Reports

The ‘social gradient’ in primary liver cancer in France: A national observational study

Authors

Marie Strigalev, David Fuks, Sandrine Katsahian, …, Alix Dhote, Vincent Mallet, Stylianos Tzedakis

Correspondence

stylianos.tzedakis@aphp.fr (S. Tzedakis).

Graphical abstract

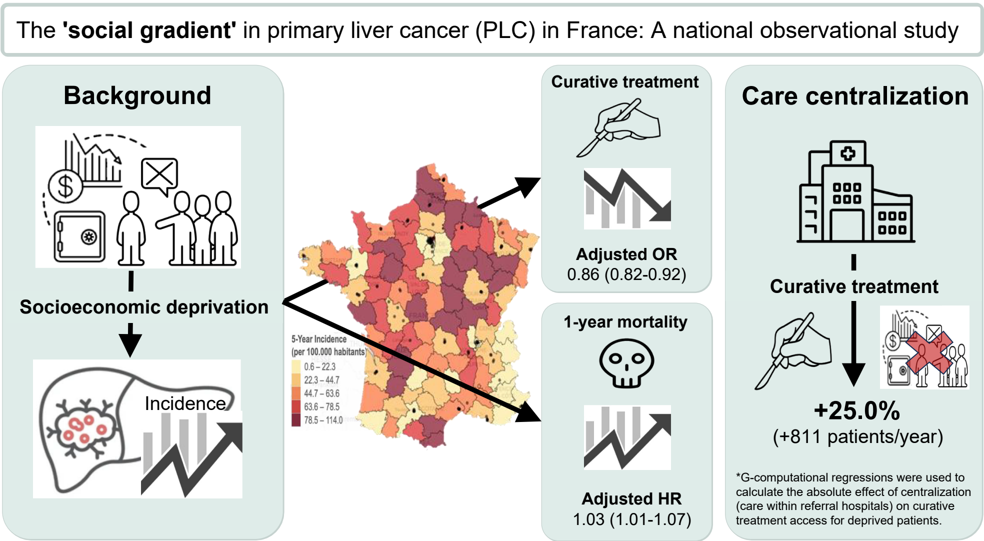

Highlights:

- Deprivation reduced access to curative treatment for primary liver cancer in France.
- Centralization of care mitigated the negative effects of social deprivation.
- Referral care could raise curative access by 25% in deprived patients.
- Findings support nationwide centralization to reduce treatment inequalities.

Impact and implications:

Social deprivation is known to increase the risk of developing primary liver cancer (PLC) and to reduce survival. However, it has been unclear if this is due to late-stage diagnoses or limited access to treatments, as earlier studies mostly considered factors like race, ethnicity, sex, and insurance status. Our study highlighted that socially deprived patients with PLC in France face reduced access to curative and palliative treatments, resulting in lower overall survival rates. A key finding was that the negative impact of social deprivation was mitigated by care centralization – when patients were diagnosed or treated in referral centers, social deprivation no longer influenced access to curative treatments or mortality outcomes. These findings support the need for centralized PLC management strategies across the country to improve care outcomes for socioeconomically disadvantaged individuals.

https://doi.org/10.1016/j.jhepr.2025.101585

© 2025 The Author(s). Published by Elsevier B.V. on behalf of European Association for the Study of the Liver (EASL). This is an open access article under the CC BY license (http://creativecommons.org/licenses/by/4.0/).

The 'social gradient' in primary liver cancer in France: A national observational study

Marie Strigalev[1,2], David Fuks[1,2], Sandrine Katsahian[2,3,4], Lucia Parlati[2,5], Ugo Marchese[1,2], Maria Conticchio[1], Charlotte Ronde-Roupie[1,2], Alexandra Nassar[1,2], Alix Dhote[1,2], Vincent Mallet[2,5], Stylianos Tzedakis[1,2,3,*]

JHEP Reports **2025**. vol. 7 | 1–8

Check for updates

Background & Aims: Social deprivation has been associated with primary liver cancer (PLC); however, its impact on access to curative treatment and survival remains uncertain. We assessed the effect of deprivation on healthcare access and evaluated whether care centralization could improve PLC management at a national level.

Methods: We conducted a retrospective longitudinal cohort study using the French National Discharge Database (2017–2021), including all adult patients with PLC. Deprivation was the primary exposure. Primary and secondary outcomes were access to curative treatment (surgery, transplantation, or ablation) and mortality. Associations were assessed using adjusted odds ratios (aORs) and hazard ratios (aHRs) derived from random-effects logistic and Cox models, clustered by French regional departments. G-computation was applied to estimate the absolute effect of centralization (treatment within referral hospitals) on curative treatment access among deprived patients.

Results: Among 62,351 patients (median age [IQR], 71 [63–78] years; 70.8% male), 45% (n = 27,872) were classified as deprived. Deprivation was associated with reduced access to curative treatment (aOR 0.89; 95% CI 0.85–0.92; *p* <0.001) and higher mortality (aHR 1.03; 95% CI 1.01–1.05; *p* <0.001). These associations were not observed among patients treated in referral hospitals (aOR 1.03; 95% CI 0.98–1.09; aHR 1.02; 95% CI 0.98–1.06). Improving access to referral hospitals was estimated to increase the probability of receiving curative treatment by 25% (95% CI 24–26), potentially benefiting 811 deprived patients per year (range, 730–895).

Conclusions: Deprivation reduced access to curative treatment and increased mortality among patients with primary liver cancer in France. Care centralization could help mitigate these disparities and improve outcomes.

© 2025 The Author(s). Published by Elsevier B.V. on behalf of European Association for the Study of the Liver (EASL). This is an open access article under the CC BY license (http://creativecommons.org/licenses/by/4.0/).

Introduction

Primary liver cancer (PLC) was the sixth most diagnosed cancer and the third leading cause of cancer-related death worldwide in 2020, with approximately 906,000 new cases and 830,000 deaths.[1] Hepatocellular carcinoma (HCC) is the most common histological subtype, accounting for 75%–85% of PLC cases.[1,2]

Among PLC risk factors, low socioeconomic status is associated with lower 5-year survival rates, as evidenced by GLOBOCAN 2018,[3] corroborating previous findings from the CONCORD-3 study, which showed higher 5-year survival rates in developed countries compared to developing ones.[4] This disparity is likely multifactorial, encompassing material conditions (*e.g.* financial resources, living and working environments), psychosocial conditions (*e.g.* social support, coping mechanisms), health behaviors (*e.g.* alcohol and tobacco use, physical activity, and diet), and access to healthcare (*e.g.* social coverage, proximity to healthcare facilities, utilization of preventive and treatment services).[5]

While the influence of social deprivation on PLC incidence and survival is well-documented, including in France,[5–8] its role in accessing curative treatment – the only chance for long-term survival – remains unclear. In the United States, deprivation has been identified as a strong predictor of mortality in patients with HCC.[9,10] However, it is unclear whether this increased mortality results from late-stage diagnoses or limited access to curative therapies, as prior studies[9,10] did not account for HCC-specific risk factors, tumor staging, or treatment modalities, focusing instead on individual determinants such as race, ethnicity, sex, marital status, and insurance type.

Unlike the United States, where healthcare access is often tied to insurance policies, the entire French population (67.5 million in 2021) has equal rights to universal, tax-financed healthcare services.[11,12] Thus, the causes of health inequalities in France are likely different and warrant investigation. If deprivation is found to hinder access to curative treatments, efforts limited to cancer prevention and screening programs may be insufficient to improve survival

* Corresponding author. Address: Department of Digestive, Hepato-biliary and Endocrine Surgery, Cochin Hospital, AP-HP, Université Paris Cité, 27, rue du Faubourg Saint Jacques, 75014, Paris, France; tel.: +33 1 58 41 17 37; fax: +33 1 58 41 29 58.
E-mail address: stylianos.tzedakis@aphp.fr (S. Tzedakis).
https://doi.org/10.1016/j.jhepr.2025.101585

outcomes in this high-risk population.[10] This is particularly pertinent for intrahepatic cholangiocarcinoma (iCCA), where screening is limited to highly selected populations with known risk factors (*e.g.* primary sclerosing cholangitis, Caroli's syndrome).[13]

Our recent findings indicated that improving access to expert centers could enhance curative treatment rates and survival for patients with cholangiocarcinoma.[14] This strategy may hold promise for addressing disparities in care among socially deprived patients. The aim of this study was to evaluate the effect of social deprivation on access to curative treatment and mortality in patients with PLC at a national level and to assess the potential role of care centralization in mitigating these disparities.

Patients and methods

Data source and design

The data source was the French National Hospital Discharge database ('*Programme de Médicalisation des Systèmes d'Information'*, PMSI), a unique large national database which contains all public and private claims for all acute inpatient/day case hospital admissions in France. During the observational period, all residents of France (67.9 million in 2022) had equal access to universal, tax-financed healthcare services. The database provided anonymized, standardized discharge summaries with patient demographics (age, sex, postal code of residency), primary and associated discharge diagnosis codes based on ICD-10, medical procedures performed (including surgery), discharge dates, length of stay, and entry and discharge modes, including in-hospital mortality.

To analyze patient trajectories and observe disease progression over time, each patient's previous and underlying conditions were traced back to 2012 using unique anonymous identifiers.[15] The coding system used in the French PMSI demonstrated 100% specificity for hard outcomes, such as cancer.[16,17] The study was approved by the '*Institut national des données de santé*' (INDS, registration number 917240) and authorized by the '*Commission nationale de l'informatique et des libertés'* (CNIL, registration number DR-2017-404). The requirement for informed consent was waived because deidentified data were used.

Study population

All patients with a PLC (ICD-10: C22.0 and C22.1) between January 1, 2012 and December 31, 2021 were analyzed. To analyze incident PLC cases, we included only patients after a washout period of 5 years, *i.e.* having a first diagnosis of PLC after January 1, 2017. Patients below 18 years old and patients with missing deprivation index, due to permanent residency outside metropolitan France (mainland France and Corsica), were excluded.

Outcome measures and exposures

The primary outcome was curative treatment for PLC and the secondary outcome was in-hospital mortality. Curative treatment included liver resection, liver transplantation and radiofrequency/microwave ablation. Palliative treatment included systemic chemotherapy, radiotherapy or locoregional treatments (intra-arterial radio- or chemoembolization) and palliative care concerned best supportive care with no active treatment. The main exposure was the socioeconomic patient status analyzed according to the French ecological deprivation index (FDep). The FDep is calculated on the municipal scale using population data taken from INSEE (the French national institute for statistics and economic studies) census records, a strong predictor of mortality related to spatial disparities in socioeconomic level and health access.[18] It is constructed from four standardized variables at the municipal level: (i) unemployment rate, (ii) proportion of manual workers, (iii) level of educational attainment, and (iv) median household income per consumption unit and is standardized at the nationwide level between quintile 1 (high-income patients) and quintile 5 (low-income patients). Quintile 4 and 5 represented the deprivation group in our study. While this terminology is not absolute, it reflects a relative gradient of socioeconomic disadvantage rather than an absolute dichotomy. Other exposures were age, sex, alcohol use disorders (defined as the presence of an alcohol-related liver disease, alcohol-related extrahepatic disease, *i.e.* chronic pancreatitis, or behavioral disorders due to former or current harmful use of alcohol),[19] smoking habits, patient comorbidities, the Charlson comorbidity index (CCI), a weighted index to predict 1-year mortality, ranging from mild (<1) to moderate (1-3) and severe (>3),[20] presence of cirrhosis, advanced-stage PLC (defined as PLC with any of decompensated cirrhosis, obstructive jaundice, ascites, portal vein thrombosis, hepatic encephalopathy or portal hypertension bleeding), late-stage cancer disease (disease with hepatic or extrahepatic metastasis at diagnosis) and French departmental healthcare services. The latter were analyzed in terms of hospital type, departmental medical density and patients' healthcare proximity. Referral hospitals were considered academic/teaching hospitals and private non-for-profit cancer centers, while private (for-profit) clinics and general regional hospitals were considered non-referral hospitals. To account for patients' healthcare proximity, the distance between the patients' geographic zone of residency (corresponding to the residence postal code entered in the PMSI database) and the hospital location (recorded according to their national health facility register identification numbers) was recorded in kilometers. Finally, medical density,[21] influencing health spatial accessibility, was recorded for all 96 French departments and was classified in three percentiles: low (165 to 246 doctors per 100,000 habitants), medium (247 to 339 doctors per 100,000 habitants) and high (339 to 503 doctors per 100,000 habitants). The code dictionary is detailed in Table S1.

Statistical analysis

Associations of deprivation with curative treatment access and mortality were estimated using multivariable binomial logistic and Cox proportional hazards regression models and associations were expressed as odds ratios (ORs) and hazard ratios (HRs) associated with their 95% CIs, respectively. Models were adjusted for age, sex, general and liver-related comorbidities, CCI, advanced PLC stage, late-stage disease, tumor histology, deprivation and Cox models were further adjusted for type of treatment. All models also included random effects for the 96 French regional departments to account for clustering of outcomes within hospital referral regions and robust standard errors were calculated. To account for potential residual confounding related to the observational nature of this

study and due to strongly imbalanced patient baseline characteristics between groups, propensity score matching (PSM) (1:1 nearest neighbor matching, caliper adjusted at 0.03 without replacement) including general and liver-related comorbidities was performed. To account for other confounders influencing access to curative treatment, sensitivity analyses were performed within subgroups of patients with varying healthcare proximity and residing in regional departments with different medical densities, in order to disentangle these effects from the impact of social deprivation. Sensitivity analyses of the deprivation effect were also performed within each FDep quintile, as well as within the subgroup of patients diagnosed and treated inside and outside referral centers, to test the effect of deprivation on curative treatment access in the presence and absence of centralization, respectively.[14] If an interaction between centralization and deprivation was found, an *ad hoc* analysis to estimate the effect of centralization on curative treatment access for deprived patients was conducted. The absolute effect of centralization was calculated through multivariable g-computational regression models, since ORs, as relative measures of effect, are difficult to interpret (especially for policy makers when estimating the absolute benefit of centralization).[22] Briefly, G-computation is a causal inference regression-based approach to estimating the average exposure effect by simulating the potential outcomes (*i.e.* curative treatment access) of individuals under different exposures (*i.e.* being treated in a referral or in a non-referral center in this study). Using this approach, a multivariable random-effects parametric regression model was used, and the predicted outcome (*i.e.* curative treatment access) was estimated for each individual as if they had been exposed (patient treated in a referral center) and unexposed (patient treated in a non-referral center). The difference between these two counterfactual outcome probabilities was the expected increase in the probability of accessing curative treatment due to being treated in a referral center rather than in a non-referral center. We then estimated the mean difference of accessing curative treatment across all deprived patients treated in non-referral centers. Multiplying the average increase of the probability to access curative treatment by the number of deprived patients treated in non-referral centers and then dividing by the number of years of the study period allows one to estimate the number of deprived patients per year that would access curative treatment in case of centralization. Bootstrap methods (500 resamples) were used to estimate accurate 95% CIs for the number of patients accessing curative treatment due to centralization. All p values presented were for a two-sided test and the threshold of significance was set at p <0.05. Multiple comparisons were adjusted with the false discovery rate (fdr) method. All analyses were conducted using R statistical software (Version 4.4.0, GUI 1.79, Big Sur ARM build – *Puppy Cup*).

Results

Patient characteristics

Between 2017 and 2021, 63,896 patients received a first diagnosis of PLC in the PMSI database. Of these, 1,471 (2.3%) had an unknown deprivation index in their discharge summaries and were excluded from the analysis. The final study population comprised 62,351 patients, with 27,872 (44.7%) classified as deprived (patient flowchart is shown in Fig. S1).

Overall, deprived patients had higher prevalences of general and liver-related comorbidities compared to non-deprived patients, including obesity (26.8% *vs.* 22.9%; p <0.001), metabolic syndrome (14.2% *vs.* 11.9%; p <0.001), diabetes (39.0% *vs.* 36.5%; p <0.001), chronic obstructive pulmonary disease (12.9% *vs.* 11.9%; p <0.001), decompensated cirrhosis (32.6% *vs.* 30.5%; p <0.001), and alcohol use disorders (39.5% *vs.* 35.6%; p <0.001). Deprived individuals had also a higher CCI (p <0.001). In contrast, chronic viral hepatitis was more frequently diagnosed in non-deprived patients (11.0% *vs.* 8.6%, p <0.001).

Although deprived patients presented a more advanced PLC at diagnosis (54.5% *vs.* 52.6%, p <0.001), there was no significant difference in the rate of late-stage disease at diagnosis between the two groups (12.9% *vs.* 13.3%, p = 0.14).

Finally, deprived patients were less often diagnosed and less frequently treated in referral centers (39.9% *vs.* 47.0%; p <0.001 and 42.3% *vs.* 50.4%; p <0.001, respectively), their residency-hospital distance was longer (29 km [8.9-62] *vs.* 15 km [6.4-31]; p <0.001) and their corresponding department medical density was lower (295 [237.0, 357] *vs.* 355 [267.0, 413], p <0.001). Other characteristics are detailed in Table 1.

Associations with access to curative treatment and mortality

Curative treatment, comprising 9,542 (15.3%) liver resections, 1,579 (2.5%) liver transplantations, and 5,625 (9.0%) radiofrequency/microwave ablations, was more commonly provided to non-deprived individuals compared to deprived individuals (28.2% *vs.* 25.0%, p <0.001). Similarly, palliative treatments were administered more frequently in non-deprived individuals (31.1% *vs.* 29.9%, p <0.001), while palliative care without active tumor-directed therapy was more frequently recorded among deprived individuals (46.0% *vs.* 40.6%, p <0.001). Multiple pairwise comparison analyses revealed that deprivation was significantly associated with lower odds of receiving curative treatment compared to palliative treatment (fully adjusted OR 0.93, 95% CI 0.88, 0.99, p_{fdr} = 0.004), as well as compared to palliative care (fully adjusted OR 0.87, 95% CI 0.83, 0.92, p_{fdr} <0.001). Deprivation also significantly reduced the odds of receiving palliative treatment (fully adjusted OR 0.91, 95% CI 0.87, 0.95, p_{fdr} <0.001). Other univariable and multivariable associations with curative and palliative treatment are detailed in Tables S2 and S3. The geographical distributions of deprivation incidence and access to curative treatment are shown in Fig. 1A,B (interactive Fig. S2A and B gives more geo-epidemiological details), respectively. Although deprivation was unevenly distributed nationally, there was no significant linear correlation between the level of deprivation in each regional department and access to curative treatment (Fig. S3).

Median (IQR) follow-up of patients was 12.0 months (2.1–39.1 months) and varied across treatment groups: 46.2 months (23.3–59.3) for patients who received curative treatment, 15.0 months (7.0–30.0) for those receiving palliative treatment, and only 2.0 months (0.6–7.2) for patients receiving palliative care. The median survival (95% CI) probability was 10.1 months (10.0–10.7 months) and 13.0 months (12.2–13.1

Table 1. Characteristics of patients with primary liver cancer by deprivation group between 2017 – 2021 in France.

Characteristic	Overall, N = 62,351[1]	No deprivation, n = 34,479 (55%)[1]	Deprivation, n = 27,872 (45%)[1]	*p* value[2]
**General risk factors**				
Age, years	71 (63.0, 78)	71 (63.0, 78)	71 (63.0, 78)	0.5
Male sex	44,138 (70.8%)	24,238 (70.3%)	19,900 (71.4%)	**0.003**
Obesity	15,371 (24.7%)	7,911 (22.9%)	7,460 (26.8%)	**<0.001**
Metabolic syndrome	8,068 (12.9%)	4,098 (11.9%)	3,970 (14.2%)	**<0.001**
Alcohol use disorders	23,285 (37.3%)	12,279 (35.6%)	11,006 (39.5%)	**<0.001**
Past or current smoker	11,683 (18.7%)	6,363 (18.5%)	5,320 (19.1%)	**0.045**
Ischemic heart disease	7,380 (11.8%)	4,015 (11.6%)	3,365 (12.1%)	0.10
Chronic kidney disease	4,495 (7.2%)	2,469 (7.2%)	2,026 (7.3%)	0.6
Chronic obstructive pulmonary disease	7,679 (12.3%)	4,095 (11.9%)	3,584 (12.9%)	**<0.001**
Diabetes mellitus	23,438 (37.6%)	12,572 (36.5%)	10,866 (39.0%)	**<0.001**
CCI				**<0.001**
Mild	15,103 (24.2%)	8,707 (25.3%)	6,396 (22.9%)	
Moderate	19,283 (30.9%)	10,673 (31.0%)	8,610 (30.9%)	
Severe	27,965 (44.9%)	15,099 (43.8%)	12,866 (46.2%)	
**Liver-related risk factors**				
Chronic viral hepatitis	6,195 (9.9%)	3,788 (11.0%)	2,407 (8.6%)	**<0.001**
Non-viral, non-metabolic, chronic liver disease	3,652 (5.9%)	2,026 (5.9%)	1,626 (5.8%)	0.8
Decompensated cirrhosis	19,601 (31.4%)	10,513 (30.5%)	9,088 (32.6%)	**<0.001**
**Cancer-related risk factors**				
Advanced PLC	33,317 (53.4%)	18,132 (52.6%)	15,185 (54.5%)	**<0.001**
Late-stage disease	8,188 (13.1%)	4,590 (13.3%)	3,598 (12.9%)	0.14
Liver cancer histology				**0.002**
HCC	41,460 (66.5%)	22,744 (66.0%)	18,716 (67.1%)	
iCCA	20,891 (33.5%)	11,735 (34.0%)	9,156 (32.9%)	
Curative treatment	16,694 (26.8%)	9,738 (28.2%)	6,956 (25.0%)	**<0.001**
Palliative treatment	18,821 (30.2%)	10,740 (31.1%)	8,081 (29.0%)	**<0.001**
Palliative care	26,836 (43.0%)	14,001 (40.6%)	12,835 (46.0%)	**<0.001**
**Healthcare-related risk factors**				
Type of hospital at PLC diagnosis				**<0.001**
Referral	27,349 (43.9%)	16,220 (47.0%)	11,129 (39.9%)	
Non-referral	35,002 (56.1%)	18,259 (53.0%)	16,743 (60.1%)	
Type of hospital at first PLC treatment				**<0.001**
Referral	29,178 (46.8%)	17,382 (50.4%)	11,796 (42.3%)	
Non-referral	33,171 (53.2%)	17,096 (49.6%)	16,075 (57.7%)	
Residency – Hospital distance at diagnosis (km)	19 (7.0, 45)	15 (6.4, 31)	29 (8.9, 62)	**<0.001**
Medical density (per 100,000 habitants)	334 (250.0, 386)	295 (237.0, 357)	355 (267.0, 413)	**<0.001**

The ecological deprivation index used in this study was the French deprivation index (Fdep), standardized into quintiles, with deprivation defined as Q4–Q5. Advanced PLC was PLC with any of the following: decompensated cirrhosis, obstructive jaundice, ascites, portal vein thrombosis, hepatic encephalopathy, or portal hypertension bleeding. Late-stage cancer was defined as PLC with hepatic or extrahepatic metastases at diagnosis. Alcohol use disorders were identified by the presence of alcoholic liver disease, alcohol-related extrahepatic disease (*e.g.* chronic pancreatitis), or behavioral disorders due to former or current harmful alcohol use. Non-metabolic liver risk factors included congenital malformations, Wilson's disease, hemochromatosis, Budd-Chiari syndrome, primary biliary cholangitis, chronic cholangitis, and autoimmune liver diseases. Metabolic syndrome was defined as type 2 diabetes with obesity and either hypertension or hyperlipidemia. The CCI, a weighted predictor of 1-year mortality, was categorized into mild (<1), moderate (1-3) and severe (>3) without including age at PLC diagnosis and liver disease severity, with higher scores indicating greater frailty. Referral hospitals included academic hospitals and private non-profit cancer centers, while non-referral hospitals comprised private (for-profit) clinics and general regional hospitals. CCI, Charlson comorbidity index; HCC, hepatocellular carcinoma; iCCA, intrahepatic cholangiocarcinoma; PLC, primary liver cancer.
[1]Median (IQR); n (%).
[2]Wilcoxon rank sum test; Fisher's exact test for count data.

months) for deprived and non-deprived patients, respectively (log-rank p <0.001, see Fig. 2). After adjusting for patient and disease confounders, as well as access to treatment, deprivation was associated with a significant reduction in survival (fully adjusted HR 1.03, 95% CI 1.01–1.07; p = 0.04) in the overall cohort (Table 2).

Sensitivity analyses and healthcare centralization

The negative impact of deprivation on access to curative treatments and survival remained significant after PSM (see Fig. S4 and Table S4 for covariate balancing between groups), was more pronounced for higher deprivation quintiles and independent of both patients' residency-hospital distance (p <0.001) and departmental medical density (Table 2). Mortality risk associated with deprivation also persisted independently of patients' residency-hospital distance, for patients living in a high medical density department (adjusted HR 1.05 95% CI 1.01 to 1.10, p = 0.017) but not for patients living in a medium (adjusted HR 1.04 95% CI 0.98 to 1.10, p = 0.2) or low medical density department (adjusted HR 0.98 95% CI 0.93 to 1.03, p = 0.4).

An interaction between deprivation and centralization was observed: for patients diagnosed in a referral hospital, unlike those diagnosed outside a referral hospital, deprivation did not affect access to curative treatment (adjusted OR 0.98, 95% CI 0.92–1.04; p = 0.5) or survival probability (adjusted HR 0.96, 95% CI 0.91–1.01; p = 0.09) (Table 2). Similar effects were observed for patients starting treatment within a referral hospital (adjusted OR 1.02, 95% CI 0.96 to 1.08, p = 0.5 and

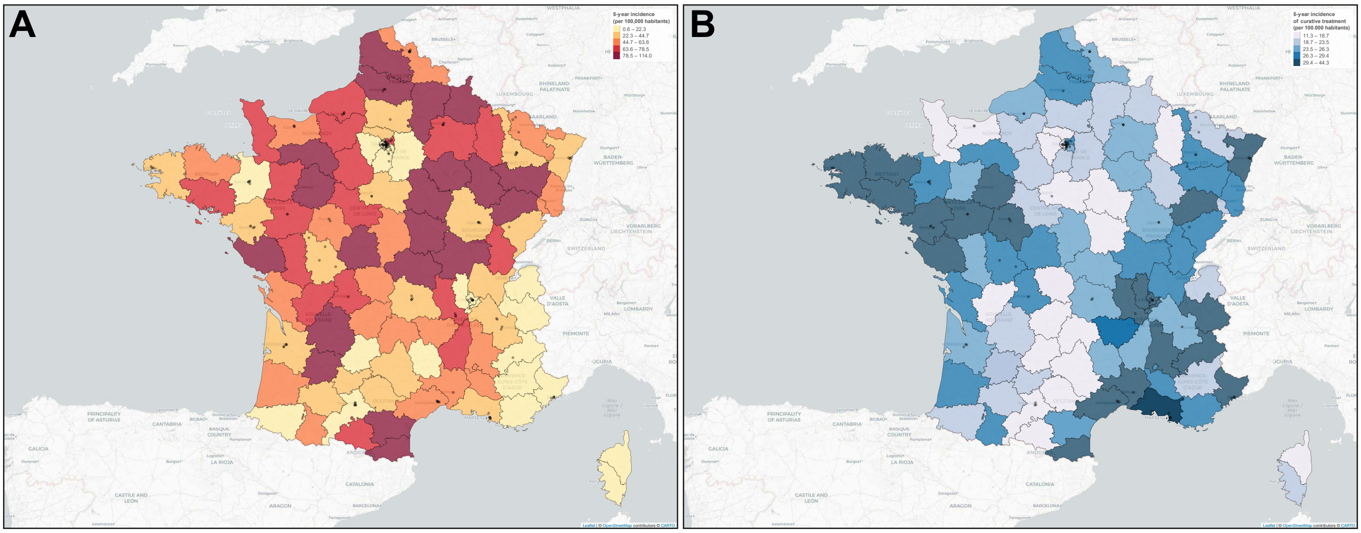

Fig. 1. Departmental variations of deprivation and curative treatment incidence among patients with primary liver cancer in France January 1, 2017 – December 31, 2021. Variations shown with regards to: (A) the 5-year incidence of social deprivation and (B) the 5-year incidence of curative treatment access. Black dots represent expert hospitals in PLC management. PLC, primary liver cancer.

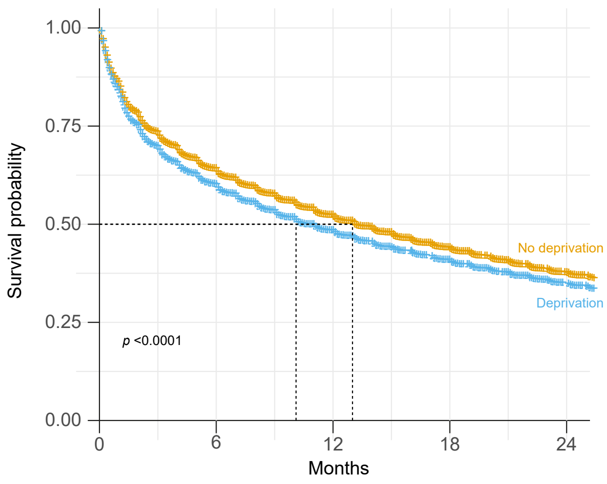

No. at risk					
No deprivation	34,479	16,250	10,872	7,557	5,250
Deprivation	27,872	12,268	8,033	5,595	3,891

Fig. 2. Probability of survival according to deprivation for patients with primary liver cancer in France January 1, 2017 – December 31, 2021. *p* values were computed with the log-rank test.

adjusted HR 0.98, 95% CI 0.93 to 1.03, p = 0.4, respectively). In the *ad hoc* analysis we estimated that centralization of PLC treatment would result in a median increase of 811 (95% CI 730 to 895) deprived patients accessing curative treatment every year, *i.e.* an average annual increase of 25.0% (95% CI 23.96 to 25.79).

Discussion

In this nationwide analysis conducted in France between 2017 and 2021, social deprivation was independently associated with reduced access to curative treatments for PLC and increased mortality. Notably, lower treatment access rates among deprived patients were not attributable to residence-hospital distance or regional medical density. A key finding was that the negative impact of social deprivation was mitigated by care centralization – when patients were diagnosed or treated in referral centers, social deprivation no longer influenced access to curative treatments or mortality outcomes.

Our study highlights the potential of care centralization to improve PLC outcomes, particularly among socioeconomically deprived individuals. This finding is consistent with evidence from England, where patients treated in tertiary centers were more likely to receive curative cancer treatments.[23] The absence of an association between residency-hospital distance or regional doctor density and access to curative PLC treatments or mortality underscores the multifaceted influence of socioeconomic factors on treatment accessibility – factors that extend beyond geographical and logistical constraints. While increased distance has been associated with poorer survival outcomes in certain cancers,[24] our results emphasize the importance of addressing intrinsic socioeconomic barriers rather than focusing solely on logistical challenges. Although late-stage diagnosis is common in PLC,[25] our analyses did not identify an association between deprivation and late-stage presentation, which may be attributable to France's universal healthcare system, ensuring equal healthcare access regardless of socioeconomic status. Although social deprivation consistently correlates with poorer cancer outcomes, its precise role in PLC remains uncertain. Previous studies, primarily from the US, demonstrated a strong link between deprivation and PLC mortality but often overlooked tumor staging and treatment access. In these US studies, deprived patients frequently presented with more advanced PLC, likely reflecting more severe underlying chronic liver disease associated with high-risk health behaviors.[5] Indeed, deprivation is closely linked to alcohol use disorders,[26] and patients with alcohol-related HCC were less likely to receive curative interventions compared to those with HCC linked to viral hepatitis in France.[27] However in our analyses, alcohol use disorders were

Table 2. Socioeconomic deprivation effect on the curative treatment access and mortality among patients with primary liver cancer in France (2017–2021).

	Access to curative treatments		Mortality	
	aOR (95% CI)[1]	*p* value	aHR (95% CI)[2]	*p* value
Overall population	0.89 (0.85, 0.92)	**<0.001**	1.03 (1.01, 1.07)	**0.04**
After PSM	0.88 (0.84, 0.91)	**<0.001**	1.03 (1.00, 1.07)	**0.04**
**Stratified by French deprivation index quintiles**				
Q1	Ref		Ref	
Q2	0.98 (0.91, 1.05)	0.5	1.03 (1.01, 1.09)	**0.018**
Q3	0.92 (0.85, 0.99)	**0.022**	1.04 (1.00, 1.08)	**0.046**
Q4	0.85 (0.79, 0.92)	**<0.001**	1.05 (1.01, 1.08)	**0.016**
Q5	0.82 (0.78, 0.91)	**<0.001**	1.08 (1.04, 1.12)	**<0.001**
**Stratified by residency-hospital distance (median)**				
<20 km	0.73 (0.67, 0.78)	**<0.001**	1.06 (1.00, 1.13)	**0.05**
≥20 km	0.83 (0.78, 0.88)	**<0.001**	1.06 (1.02, 1.11)	**0.004**
**Stratified by departmental medical density (IQR)**				
Low	0.81(0.74, 0.89)	**<0.001**	0.98 (0.93, 1.03)	0.4
Medium	0.93 (0.86, 0.99)	**0.05**	1.04 (0.98, 1.10)	0.2
High	0.87 (0.81, 0.92)	**<0.001**	1.05 (1.01, 1.10)	**0.017**
**Stratified by type of hospital center at diagnosis**				
Referral	0.98 (0.92, 1.04)	0.5	0.96 (0.91, 1.01)	0.09
Non-referral	0.79 (0.74, 0.85)	**<0.001**	1.05 (1.02, 1.09)	**0.003**
**Stratified by type of hospital center at first care**				
Referral	1.02 (0.96, 1.08)	0.5	0.98 (0.93, 1.03)	0.4
Non-referral	0.75 (0.70, 0.81)	**<0.001**	1.04 (1.01, 1.08)	**0.01**

[1]The probability of receiving curative treatments (liver resection, transplantation or ablation) was estimated using a random-effects multivariable generalized linear model, adjusted for age, sex, general and liver-related comorbidities, CCI, advanced PLC stage, late-stage disease, tumor histology, and deprivation.
[2]Mortality risks were assessed using multivariable Cox proportional hazards models, stratified by the same variables and treatment type (curative or palliative). aHR, adjusted hazard ratio; aOR, adjusted odds ratio; CCI, Charlson comorbidity index; HCC, hepatocellular carcinoma; iCCA, intrahepatic cholangiocarcinoma; PLC, primary liver cancer; PSM, propensity score matching.

not directly associated with decreased access to curative treatments, suggesting that these disorders are not the primary drivers of treatment disparities observed elsewhere, such as reduced access to organ support during the COVID-19 pandemic.[28]

Health inequalities are still observed in France, without a decrease over time, and are associated with chronic diseases (cardiovascular, respiratory or metabolic) including several types of cancer (stomach, liver, lips–mouth–pharynx and lung cancer).[5,29] In the US, an observational study has shown that social deprivation on an area-level had a negative effect on survival in patients with non-metastatic lung, prostate and colorectal cancer.[30] Similarly, in 2023, Nephew *et al.* studied the effect of social deprivation at individual and neighborhood levels on HCC and iCCA mortality in Indiana.[10] They reported that social deprivation at the neighborhood level, estimated by the Social Deprivation Index, and specific individual-level factors (*e.g.* lack of health insurance, never having been married) were associated with worse survival outcomes for HCC but not for iCCA. These findings align with our study, which demonstrates that social deprivation – estimated using the FDep index at the neighborhood level –reduces access to curative treatments and increases overall PLC mortality.

Despite its strengths, including a nationwide, multicenter design and a large sample size, our study has limitations. Its retrospective nature makes it susceptible to residual confounding despite the use of robust adjustment strategies and caution is warranted when interpreting the findings, as we could not adjust for all patient-level confounders (*e.g.* psychosocial, medical, or behavioral factors) not captured by the deprivation index. Additionally, the FDep index may not fully capture individual-level socioeconomic hardship and may lead to exposure misclassification, particularly in socioeconomically mixed urban areas. Furthermore, although using a France-specific index might limit interpretation of our findings, notably due to the inability to study the individual components of this index, the FDep index reflects a relative deprivation score rather than an absolute socioeconomic measure, facilitating the extrapolation of our observations to other settings where comparable indices exist and making the equity-focused implications of our findings relevant across health-care systems.[31] We also excluded patients with an unknown FDep index (*e.g.* those with permanent residence outside metropolitan France), which may have introduced bias, although this subgroup represented only a small proportion (2.3%) of the cohort.

A further limitation of our study is the absence of race and ethnicity data, which are known to intersect with socioeconomic status in shaping health outcomes; however, in accordance with French legal and ethical standards, such data are not collected in administrative health databases, including the PMSI. Moreover, although this study can claim representativeness of patients in French hospitals, there may be cases of PLC and deaths outside of hospitals. Nevertheless, patients with PLC in France are hospitalized at least once, either for diagnosis, biopsy, or treatment initiation, and fewer than 5% of PLC deaths occurred outside hospitals during the study period.[32,33] On the other hand, although our study may not capture long-term oncologic outcomes or recurrence, the observed differences in follow-up durations across treatment groups – particularly the markedly shorter trajectories in patients receiving palliative treatment and care – highlight the early prognostic consequences of unequal treatment access. Finally, iCCA incidence was reported to be higher in this study and is most likely related to a well-established 5–15% overlap

between HCC and iCCA, as well as modern challenges associated with the ICD-10 coding system in accurately distinguishing between HCC and iCCA.[34] Nevertheless, our findings on deprivation are rather unlikely to have been influenced by this misclassification, since HCC and iCCA were considered together.

In summary, our study highlights that socially deprived patients with PLC in France face reduced access to curative treatments, resulting in lower overall survival rates. These findings support the need for centralized PLC management strategies across the country to improve care outcomes for socioeconomically disadvantaged individuals.

Affiliations

[1]*AP-HP.Centre, Groupe Hospitalier Cochin Port Royal, DMU Cancérologie et Spécialités Médico-chirurgicales, Service de Chirurgie Digestive, Hépatobiliaire et Endocrinienne, Paris, France;* [2]*Université Paris Cité, F-75006, Paris, France;* [3]*INSERM, UMR 1138, Centre de Recherche des Cordeliers, Centre Inria de Paris, Équipe HeKA, France;* [4]*AP-HP, Hôpital Européen Georges-Pompidou, Service D'Épidémiologie et de Biostatistiques, Paris, France;* [5]*AP-HP.Centre, Université Paris Centre, Groupe Hospitalier Cochin Port Royal, DMU Cancérologie et Spécialités Médico-chirurgicales, Service des Maladies Du Foie, Paris, France*

Abbreviations

CCI, Charlson comorbidity index; FDep, French ecological deprivation index; FDR, false discovery rate; HCC, hepatocellular carcinoma; iCCA, intrahepatic cholangiocarcinoma; PLC, primary liver cancer; PMSI, *Programme de Médicalisation des Systèmes d'Information*.

Financial support

Authors did not receive any specific grant from funding agencies in the public, commercial, or not-for-profit sectors for this research.

Conflict of interest

The authors have no conflicts of interest to disclose.

Please refer to the accompanying ICMJE disclosure forms for further details.

Authors' contributions

(I) Study conception and design: ST, DF, VM, AN, SK; (II) Acquisition of data, analysis and interpretation: ST, VM, SK, LP, MC, CRR; (III) Manuscript drafting: MC, CRR, AN, UM, SK; (IV) Critical revision of the manuscript for important intellectual content: All authors. (V) Statistical analysis: ST, VM, UM, AN, DF; (VI) Study supervision: DF, VM, SK. (VI) Final approval of manuscript: All authors.

Data availability

The data that support the findings of this study are available from *Agence Technique d Information Hospitalière* but restrictions apply to the availability of these data, which were used under license for the current study and therefore are not publicly available. Data dictionary or other specified related documents (*e.g.* study protocol, statistical analysis plan) can be available on demand after approval of a proposal by the authors.

Supplementary data

Supplementary data to this article can be found online at https://doi.org/10.1016/j.jhepr.2025.101585.

References

[1] Sung H, Ferlay J, Siegel RL, et al. Global cancer statistics 2020: GLOBOCAN estimates of incidence and mortality worldwide for 36 cancers in 185 countries. CA Cancer J Clin 2021 mai;71(3):209–249.

[2] Rumgay H, Ferlay J, de Martel C, et al. Global, regional and national burden of primary liver cancer by subtype. Eur J Cancer Oxf Engl 1990. 2022 Jan;161:108–118.

[3] Sharma R. Descriptive epidemiology of incidence and mortality of primary liver cancer in 185 countries: evidence from GLOBOCAN 2018. Jpn J Clin Oncol 2020 Dec 16;50(12):1370–1379.

[4] Allemani C, Matsuda T, Di Carlo V, et al. Global surveillance of trends in cancer survival 2000–14 (CONCORD-3): analysis of individual records for 37 513 025 patients diagnosed with one of 18 cancers from 322 population-based registries in 71 countries. The Lancet 2018 Mar;391(10125):1023–1075.

[5] Bryere J, Dejardin O, Launay L, et al. Socioeconomic status and site-specific cancer incidence, a Bayesian approach in a French Cancer Registries Network study. Eur J Cancer Prev 2018 Jul;27(4):391.

[6] Marant-Micallef C, Shield KD, Vignat J, et al. Nombre et fractions de cancers attribuables au mode de vie et à l'environnement en France métropolitaine en 2015 : résultats principaux. Bull Epidémiol Hebd 2018;(21):442–448. http://invs.santepubliquefrance.fr/beh/2018/21/2018_21_2.html.

[7] Bryere J, Dejardin O, Bouvier V, et al. Socioeconomic environment and cancer incidence: a French population-based study in Normandy. BMC Cancer 2014 Feb 13;14:87.

[8] Tron L, Belot A, Fauvernier M, et al. Socioeconomic environment and disparities in cancer survival for 19 solid tumor sites: an analysis of the French Network of Cancer Registries (FRANCIM) data. Int J Cancer 2019;144(6):1262–1274.

[9] Oluyomi AO, Mohammadi KA, El-Serag HB, et al. Mediating effects of neighborhood-level socioeconomic deprivation on the association between race/ethnicity and advanced hepatocellular carcinoma. Cancer Epidemiol Biomarkers Prev 2022 Jul 1;31(7):1402–1409.

[10] Nephew LD, Gupta D, Carter A, et al. Social determinants of health impact mortality from HCC and cholangiocarcinoma: a population-based cohort study. Hepatol Commun 2023 Feb 9;7(3):e0058.

[11] Butler DC, Petterson S, Phillips RL, et al. Measures of social deprivation that predict health care access and need within a rational area of primary care service delivery. Health Serv Res 2013 Apr;48(2 Pt 1):539–559.

[12] Mackenbach JP, Stirbu I, Roskam AJR, et al. Socioeconomic inequalities in health in 22 European countries. N Engl J Med 2008 Jun 5;358(23):2468–2481.

[13] Neuzillet C, Decraecker M, Larrue H, et al. Management of intrahepatic and perihilar cholangiocarcinomas: guidelines of the French association for the study of the liver (AFEF). Liver Int Off J Int Assoc Study Liver 2024 Jul 5.

[14] Tzedakis S, Challine A, Katsahian S, et al. Clinical care pathways of patients with biliary tract cancer: a French nationwide longitudinal cohort study. Eur J Cancer 2024 May 1;202:114018.

[15] Agence Technique de l'Information sur l'Hospitalisation. Aide à l'utilisation des informations de chaînage [How to use de-identified patient information] Lyon, France: Agence technique de l'information sur l'hospitalisation. 2014 [updated October 6, 2015, http://www.atih.sante.fr/aide-lutilisation-des-informations-de-chainage.

[16] Schwarzinger M, Baillot S, Yazdanpanah Y, et al. Contribution of alcohol use disorders on the burden of chronic hepatitis C in France, 2008–2013: a nationwide retrospective cohort study. J Hepatol 2017 Sep;67(3):454–461.

[17] Tzedakis S, Yzhaky Shapira O, Schwarzinger M, et al. COVID-19 and excess mortality of patients with liver cancer in France, January 2020–September 2022. Br J Surg 2024 Jan 3;111(1):znad359.

[18] Rey G, Jougla E, Fouillet A, et al. Ecological association between a deprivation index and mortality in France over the period 1997 – 2001: variations with spatial scale, degree of urbanicity, age, gender and cause of death. BMC Public Health 2009 Dec;9(1):33.

[19] Rehm J, Mathers C, Popova S, et al. Global burden of disease and injury and economic cost attributable to alcohol use and alcohol-use disorders. The Lancet 2009 juin;373(9682):2223–2233.

[20] Quan H, Li B, Couris CM, Fushimi K, et al. Updating and validating the Charlson comorbidity index and score for risk adjustment in hospital discharge abstracts using data from 6 countries. Am J Epidemiol 2011 Mar 15;173(6):676–682.

[21] Institut national de la statistique et des études économiques. Professionnels de santé au 1er janvier 2023. https://www.insee.fr/fr/statistiques/2012677#tableau-TCRD_068_tab1_departements. [Accessed 25 August 2024].

[22] Austin PC, Urbach DR. Using G-computation to estimate the effect of regionalization of surgical services on the absolute reduction in the occurrence of adverse patient outcomes. Med Care 2013 Sep;51(9):797–805.

[23] Vallance AE, vanderMeulen J, Kuryba A, et al. Impact of hepatobiliary service centralization on treatment and outcomes in patients with colorectal cancer and liver metastases. Br J Surg 2017 Jun;104(7):918–925.

[24] Siegel J, Engelhardt KE, Hornor MA, et al. Travel distance and its interaction with patient and hospital factors in pancreas cancer care. Am J Surg 2021 Apr;221(4):819–825.
[25] Patel N, Yopp AC, Singal AG. Diagnostic delays are common among patients with hepatocellular carcinoma. J Natl Compr Canc Netw 2015 May;13(5):543–549.
[26] Menvielle G, Kunst AE, Stirbu I, et al. Socioeconomic inequalities in alcohol related cancer mortality among men: to what extent do they differ between Western European populations? Int J Cancer 2007 Aug;121(3):649–655.
[27] Costentin CE, Sogni P, Falissard B, et al. Geographical disparities of outcomes of hepatocellular carcinoma in France: the heavier burden of alcohol compared to hepatitis C. Dig Dis Sci 2020 Jan;65(1):301–311.
[28] Mallet V, Beeker N, Bouam S, et al. Prognosis of French COVID-19 patients with chronic liver disease: a national retrospective cohort study for 2020. J Hepatol 2021 Oct;75(4):848–855.
[29] International Agency for Research on Cancer. Les cancers attribuables au mode de vie et à l'environnement en France métropolitaine. Chapitre 17 : Facteurs socio-économiques. 2018 [cited 2024 Aug 1]. Available from: https://gco.iarc.fr/includes/PAF/PAF_FR_report.pdf.
[30] Cheng E, Soulos PR, Irwin ML, et al. Neighborhood and individual socioeconomic disadvantage and survival among patients with nonmetastatic common cancers. JAMA Netw Open 2021 Dec 1;4(12):e2139593.
[31] Mogin G, Gorasso V, Baravelli CM, et al. BEST-COST project: a scoping review on the use of multiple deprivation indices in Europe. Eur J Public Health 2024 Oct 28;34(Suppl 3). ckae144.1570.
[32] Cowppli-Bony A, Colonna M, Ligier K, et al. [Descriptive epidemiology of cancer in metropolitan France: incidence, survival and prevalence]. Bull Cancer 2019;106(7–8):617–634.
[33] Santé Publique France. National estimates of cancer incidence and mortality in metropolitan France between 1990 and 2018: Study based on the French network of cancer registries, Francim [Estimations nationales de l'incidence et de la mortalité par cancer en France métropolitaine entre 1990 et 2018: Étude à partir des registres des cancers du réseau Francim]. 2019. https://www.e-cancer.fr/content/download/266450/3759432/file/Synthese_Estimationsnationalesincidenceetmortaliteparcancer_juillet_2019.pdf. [Accessed 26 June 2025].
[34] Khan SA, Genus T, Morement H, et al. Global trends in mortality from intrahepatic and extrahepatic cholangiocarcinoma. J Hepatol 2019 Dec;71(6):1261–1262.

Keywords: Socioeconomic deprivation; Primary liver cancer; Hepatocellular carcinoma; Cholangiocarcinoma; French national discharge database; Liver resection; Liver transplantation; Tumor ablation; Care centralization.
Received 1 April 2025; received in revised form 14 August 2025; accepted 18 August 2025; Available online 5 September 2025

Journal of Hepatology, Volume *7*

Supplemental information

The `social gradient' in primary liver cancer in France: A national observational study

Marie Strigalev, David Fuks, Sandrine Katsahian, Lucia Parlati, Ugo Marchese, Maria Conticchio, Charlotte Ronde-Roupie, Alexandra Nassar, Alix Dhote, Vincent Mallet, and Stylianos Tzedakis

The 'social gradient' in primary liver cancer in France:

A national observational study

Marie Strigalev, David Fuks, Sandrine Katsahian, Lucia Parlati, Ugo Marchese, Maria Conticchio, Charlotte Ronde-Roupie, Alexandra Nassar, Alix Dhote, Vincent Mallet, Stylianos Tzedakis

Table of contents

Table S1……………………………………………………………………………………2

Table S2……………………………………………………………………………………6

Table S3…………………………………………………………………………...……11

Table S4………………………………………………………………………………….. 14

Fig. S1………………………………………………………………………….……….....16

Fig. S2………………………………………………………………………….……….....17

Fig. S3………………………………………………………………………….……….....18

Fig. S4………………………………………………………………………….……….....19

**Table S1:** Code dictionary	
**Variable**	ICD-10 and CCAM medical procedure codes
**Acute liver failure**	K720, K729
**Alcohol use disorders**	E244, E511, F101, F102, F103, F104, F105, F106, F107, F108, F109, F1020, F1021, F1022, F1023, G312, G621, G721, I426, K292, K70, K852, K860, O354, Z502, Z714, Z721
**Alcoholic liver disease**	K70
**Biliary duct surgery**	HMFA010, HMFA009, HMLA001, HMLC001, HMFA001, HMFA002, HMFA005, HMFA006, HMFC003, HMFC005
**Chronic kidney disease**	E102, E112, E122, E132, E142, I151, JAHB001, JAHH002, JAHC001, JAHA001, JAHJ006, JAHJ007, N02, N04, N06, N07, N08, N181, N182, N19, N25, N083
**Chemotherapy for Cancer**	Z511, Z512
**Chemoembolization**	EDLF017, EDLF016, EDLF015, EDLF014
**Cirrhosis**	I859, I864, I982, I9829, K703, K717, K743, K744, K745, K746, K766
**Decompensated Cirrhosis**	R17, K704, K711, K72, K767, I280, R18, J948, K65, HPHB003, HPJB001, EHCA003, EHCA006, EHCA009,

	EHCA007, EHCA004, EHCA002, EHCA005, EHCA010, EHCA001, HEPA005, HEPA004, HEPA007
**Congestive heart failure**	I099, I110, I130, I132, I255, I420, I425, I426, I427, I428, I429, I43, I50, P290
**Chronic Obstructive Pulmonary Disease**	I278, I279, J40, J41, J42, J43, J44, J45, J46, J47, J60, J61, J62, J63, J64, J65, J66, J67, J684, J701, J703
**Diabetes mellitus complicated**	E102, E103, E104, E105, E107, E112, E113, E114, E115, E117, E122, E123, E124, E125, E127, E132, E133, E134, E135, E137, E142, E143, E144, E145, E147, H360, N083, H280, G632
**Diabetes mellitus uncomplicated**	E100, E101, E106, E108, E109, E110, E111, E116, E118, E119, E120, E121, E126, E128, E129, E130, E131, E136, E138, E139, E140, E141, E146, E148, E149
**Gastrectomy**	HFPA001, HFFA009, HFFA002, HFFA003, HFFA008, HFFA006, HFFA005, HFMA005, HFFC001, HFFC002, HFFC012, HFFC017
**Gastro Esophageal Varices Bleeding**	I850, I983, I9820, EHBD001, EHNE002, HESE001, HESE002
**Hepatitis B, C and D virus**	B181, B162, B169, B182, B160, B161, B180, B170

**Hepatocellular carcinoma**	C220
**Hepatopulmonary and Hepatorenal Syndrome**	I280, K767
**Hepatic Encephalopathy**	K704, K711, K74
**Intrahepatic cholangiocarcinoma**	C221
**Jaundice**	R17
**Liver biopsy**	HLHB001, HLHJ003, HLHJ006, HLHH006, HLHJ005, HLHH007, HLHH001, HLHH005
**Liver resection**	HLFA003, HLFA009, HLFA011, HLFA020, HLFA006, HLFC002, HLFC004, HLFC027, HLFC032, HLFA004, HLFA005, HLFA007, HLFA010, HLFA017, HLFA018, HLFC037, HLFC801
**Liver ablation (radiofrequency, microwave)**	HLNN900, HLNK001, HLNM001, HLNA007, HLNC003
**Liver failure**	K720, K729, K711, K763
**Liver Transplantation**	Z944, HLEA001, HGEA002, HLEA002, HGEA004

**Metastasis intra- and extra-hepatic**	C787, C780, C782, C783, C781, C784, C788, C798, C799, C793, C797, C786, C482, K669, K668, C796, C795
**Non Viral Non Metabolic Chronic Liver Disease**	E831, E84, FEJF003, FEJF006, FEJF008, E830, N163, I820, K743, K754, Q44, T864, Z944
**Obesity**	E66
**Portal Vein Thrombosis**	I81
**Portal Hypertension**	K766
**Phlebitis and Thrombophlebitis**	I80
**Palliative Care**	Z515
**Radioembolization**	EDLL001, EDLL002
**Smoking**	F17, Z716, Z720, T652

Table S2: Univariable and multivariable associations with curative treatment of PLC in France 2017 - 2021

Characteristic	Univariable Associations				Multivariable Associations	
	**Overall, N = 62,351**[1]	**Palliative treatment or palliative care, n = 45,657 (73%)**[1]	**Curative treatment, n = 16,694 (27%)**[1]	**p-value**[2]	**aOR (95% CI)**	**p-value**
**Deprivation**	27,872 (44.7%)	20,916 (45.8%)	6,956 (41.7%)	**<0.001**	0.89 (0.85, 0.92)	**<0.001**
**Advanced PLC**	33,317 (53.4%)	24,485 (53.6%)	8,832 (52.9%)	0.11	0.58 (0.55, 0.62)	**<0.001**
**Late-stage disease**	8,188 (13.1%)	7,856 (17.2%)	332 (2.0%)	**<0.001**	0.11 (0.10, 0.12)	**<0.001**
**Age, years**	71 (63.0, 78)	72 (64.0, 80)	68 (61.0, 74)	**<0.001**	0.97 (0.97,0.97)	**<0.001**

**Male Sex**	44,138 (70.8%)	31,445 (68.9%)	12,693 (76.0%)	**<0.001**	1.03 (0.99, 1.08)	0.2
**Obesity**	15,371 (24.7%)	10,348 (22.7%)	5,023 (30.1%)	**<0.001**	1.39 (1.31, 1.47)	**<0.001**
**Metabolic syndrome**	8,068 (12.9%)	5,394 (11.8%)	2,674 (16.0%)	**<0.001**	0.96 (0.88, 1.04)	0.3
**Smoking habits**	11,683 (18.7%)	7,768 (17.0%)	3,915 (23.5%)	**<0.001**	1.13 (1.08, 1.19)	**<0.001**
**Alcohol use disorders**	23,285 (37.3%)	15,674 (34.3%)	7,611 (45.6%)	**<0.001**	1.02 (0.97, 1.08)	0.4
**Ischemic heart disease**	7,380 (11.8%)	5,350 (11.7%)	2,030 (12.2%)	0.13	1.02 (0.96, 1.09)	0.5
**Chronic kidney disease**	4,495 (7.2%)	3,080 (6.7%)	1,415 (8.5%)	**<0.001**	1.10 (1.02, 1.19)	**<0.001**
**Chronic obstructive pulmonary disease**	7,679 (12.3%)	5,641 (12.4%)	2,038 (12.2%)	0.6	0.97 (0.91, 1.03)	0.4

**Diabetes**	23,438 (37.6%)	16,540 (36.2%)	6,898 (41.3%)	**<0.001**	1.17 (1.12, 1.23)	**<0.001**
**Viral hepatitis**	6,195 (9.9%)	3,649 (8.0%)	2,546 (15.3%)	**<0.001**	1.40 (1.31, 1.50)	**<0.001**
**Non viral non metabolic chronic liver disease**	3,652 (5.9%)	1,396 (3.1%)	2,256 (13.5%)	**<0.001**	3.84 (3.57, 4.14)	**<0.001**
**Cirrhosis**	21,184 (34.0%)	14,117 (30.9%)	7,067 (42.3%)	**<0.001**	1.42 (1.33, 1.51)	**<0.001**
**CCI**				**<0.001**		
Mild	15,103 (24.2%)	10,239 (22.4%)	4,864 (29.1%)		1 (Ref)	
Moderate	19,283 (30.9%)	13,965 (30.6%)	5,318 (31.9%)		0.75 (0.71, 0.80)	**<0.001**
Severe	27,965 (44.9%)	21,453 (47.0%)	6,512 (39.0%)		0.63 (0.59, 0.67)	**<0.001**

**Liver cancer histology**				**0.002**		
ICC	20,891 (33.5%)	17,582 (38.5%)	3,309 (19.8%)		Ref	
HCC	41,460 (66.5%)	28,075 (61.5%)	13,385 (80.2%)		1.86 (1.76, 1.95)	**<0.001**
**Type of hospital center at diagnosis**				**<0.001**		
Non-referral	35,002 (56.1%)	28,927 (63.4%)	6,075 (36.4%)		Ref	
Referral	27,349 (43.9%)	16,730 (36.6%)	10,619 (63.6%)		2.69 (2.57, 2.80)	**<0.001**
[1]Median (IQR); n (%)						
[2]Wilcoxon rank sum test; Fisher's Exact Test for Count Data						

Adjusted outcome was calculated using a fully adjusted mixed effects generalized linear models. Advanced PLC was PLC with any of decompensated cirrhosis, obstructive jaundice, ascites, portal vein thrombosis, hepatic encephalopathy or portal hypertension bleeding. Late-stage cancer disease was defined as PLC with hepatic or extrahepatic metastasis at diagnosis. The Charlson comorbidity index is a weighted index to predict one-year mortality in patients with multiple comorbidities and was classified in three percentiles higher scores indicating higher frailty and ranging from mild (2-6) to moderate (6-8) and severe (>8), without entering age and liver disease. The ecological deprivation index used in the study was the French deprivation index *Fdep* standardized in quintiles (Q) and deprivation was defined as Q4-Q5. Abbreviations: CCI: Charlson comorbidity index; HCC: hepatocellular carcinoma; ICC: intrahepatic cholangiocarcinoma; PLC: Primary Liver Cancer; aOR = adjusted Odds Ratio; CI = Confidence Interval

Table S3: Association of deprivation and treatment access for PLC patients in France 2017 - 2021

Characteristic	Curative treatment (vs palliative care)		Palliative treatment (vs palliative care)		Curative treatment (vs palliative treatment)	
	**aOR (95% CI)[1]**	**p-value[2]**	**aOR (95% CI)[1]**	**p-value[2]**	**aOR (95% CI)[1]**	**p-value[2]**
**Deprivation**	0.87 (0.83, 0.92)	**<0.001**	0.91 (0.87, 0.95)	**<0.001**	0.93 (0.88, 0.98)	**0.004**
**Advanced PLC**	0.52 (0.48, 0.55)	**<0.001**	0.92 (0.86, 0.97)	**0.003**	0.62 (0.58, 0.66)	**<0.001**
**Late-stage disease**	0.09 (0.08, 0.11)	**<0.001**	0.74 (0.70, 0.78)	**<0.001**	0.15 (0.13, 0.17)	**<0.001**
**Age, years**	0.63 (0.60, 0.67)	**<0.001**	0.67 (0.64, 0.70)	**<0.001**	0.83 (0.79, 0.88)	**<0.001**
**Male Sex**	1.05 (0.99, 1.11)	0.081	1.05 (1.00, 1.10)	0.062	1.00 (0.94, 1.05)	0.9
**Obesity**	1.50 (1.39, 1.61)	**<0.001**	1.25 (1.17, 1.33)	**<0.001**	1.19 (1.11, 1.28)	**<0.001**
**Metabolic syndrome**	0.93 (0.84, 1.03)	0.14	0.98 (0.89, 1.07)	0.6	0.97 (0.88, 1.07)	0.5
**Smoking habits**	1.17 (1.10, 1.25)	**<0.001**	1.16 (1.10, 1.23)	**<0.001**	1.03 (0.97, 1.09)	0.4

**Alcohol use disorders**	1.06 (0.99, 1.12)	0.082	1.04 (0.98, 1.10)	0.2	0.97 (0.92, 1.03)	0.4
**Ischemic heart disease**	1.06 (0.99, 1.15)	0.10	1.08 (1.01, 1.15)	**0.030**	0.98 (0.91, 1.05)	0.5
**Chronic kidney disease**	1.07 (0.98, 1.17)	0.15	0.98 (0.90, 1.06)	0.6	1.10 (1.00, 1.21)	**0.042**
**Chronic obstructive pulmonary disease**	0.99 (0.92, 1.06)	0.7	1.01 (0.95, 1.08)	0.7	0.96 (0.89, 1.03)	0.3
**Diabetes**	1.35 (1.27, 1.43)	**<0.001**	1.32 (1.25, 1.39)	**<0.001**	1.00 (0.94, 1.06)	>0.9
**Viral hepatitis**	1.35 (1.25, 1.46)	**<0.001**	0.89 (0.82, 0.96)	**0.003**	1.42 (1.32, 1.53)	**<0.001**
**Non viral non metabolic chronic liver disease**	3.53 (3.20, 3.89)	**<0.001**	1.12 (1.00, 1.26)	0.061	3.35 (3.04, 3.70)	**<0.001**
**Cirrhosis**	1.31 (1.22, 1.41)	**<0.001**	0.83 (0.77, 0.88)	**<0.001**	1.53 (1.43, 1.64)	**<0.001**
**CCI**						
Mild	Ref		Ref		Ref	
Moderate	0.46 (0.43, 0.50)	**<0.001**	0.39 (0.37, 0.42)	**<0.001**	1.11 (1.04, 1.18)	**0.001**

Severe	0.34 (0.32, 0.37)	**<0.001**	0.28 (0.26, 0.30)	**<0.001**	1.08 (1.00, 1.17)	**0.042**
**Liver cancer histology**						
ICC	Ref		Ref		Ref	
HCC	1.79 (1.68, 1.90)	**<0.001**	0.78 (0.75, 0.82)	**<0.001**	1.93 (1.82, 2.05)	**<0.001**
**Type of hospital center at diagnosis**						
Non-referral	Ref		Ref		Ref	
Referral	4.40 (4.19, 4.63)	**<0.001**	2.63 (2.51, 2.75)	**<0.001**	1.53 (1.46, 1.61)	**<0.001**

[1]Adjusted outcome was calculated using a random-effects generalized linear model adjusted for age, sex, general and liver-related comorbidities, CCI, advanced PLC stage, late-stage disease, tumor histology, type of hospital at diagnosis (referral or non-referral) and deprivation.

[2]p-values were adjusted with the false discovery rate method due to multiple comparisons

Abbreviations: CCI: Charlson comorbidity index; PLC: Primary Liver Cancer; aOR = adjusted Odds Ratio; CI = Confidence Interval

Table S4. General and liver-related characteristics balancing after propensity score matching for patients with primary liver cancer by deprivation group between 2017 – 2021 in France

Characteristic	No Deprivation, n = 27,872[1]	Deprivation, n = 27,872[1]	Difference (95% CI)[2]	P value[3]
**General risk factors**				
**Age, years**	71 (63, 78)	71 (63, 78)	0.00 (-0.01, 0.02)	0.202
**Age, > 70 years-old**	14,166 (51%)	14,021 (50%)	0.01 (-0.01, 0.03)	0.219
**Male Sex**	19,940 (72%)	19,900 (71%)	0.00 (-0.01, 0.02)	0.708
**Obesity**	7,398 (27%)	7,460 (27%)	-0.01 (-0.02, 0.01)	0.553
**Metabolic syndrome**	3,895 (14%)	3,970 (14%)	-0.01 (-0.02, 0.01)	0.361
**Alcohol use disorders**	10,952 (39%)	11,006 (39%)	0.00 (-0.02, 0.01)	0.640
**Past or current smoker**	5,347 (19%)	5,320 (19%)	0.00 (-0.01, 0.02)	0.771
**Ischemic heart disease**	3,323 (12%)	3,365 (12%)	0.00 (-0.02, 0.01)	0.584
**Chronic kidney disease**	2,031 (7.3%)	2,026 (7.3%)	0.00 (-0.02, 0.02)	0.935
**Chronic obstructive pulmonary disease**	3,487 (13%)	3,584 (13%)	-0.01 (-0.03, 0.01)	0.217
**Diabetes mellitus**	10,885 (39%)	10,866 (39%)	0.00 (-0.02, 0.02)	0.869
**CCI**	158 (50)		0.01 (-0.01, 0.03)	0.609
Mild	6,298 (23%)	6,396 (23%)		
Moderate	8,660 (31%)	8,610 (31%)	0.12 (0.08, 0.20)	0.4

Severe	12,914 (46%)	12,866 (46%)		
**Liver-related risk factors**				
Chronic Viral hepatitis	2,401 (8.6%)	2,407 (8.6%)	0.00 (-0.02, 0.02)	0.928
Non viral, non metabolic, chronic liver disease	1,627 (5.8%)	1,626 (5.8%)	0.00 (-0.02, 0.02)	0.986
Decompensated cirrhosis	9,138 (33%)	9,088 (33%)	0.00 (-0.01, 0.02)	0.652
Advanced PLC	15,318 (55%)	15,185 (54%)	0.01 (-0.01, 0.03)	0.258
Late-stage disease	3,545 (13%)	3,598 (13%)	-0.01 (-0.02, 0.01)	0.502
Liver cancer histology			0.00 (-0.02, 0.02)	0.993
HCC	18,715 (67%)	18,716 (67%)		
ICC	9,157 (33%)	9,156 (33%)		

[1]Median (IQR); n (%); [2]Standardized Mean Difference; [3]McNemar's test and paired t-tests for paired data

Standardized mean differences (SMDs) were then used to assess covariate balance, with an SMD ≤ 0.01 indicating negligible differences, values from 0.0101 to 0.030 indicating very small differences, values from 0.0301 to 0.050 indicating small differences, and values > 0.050 indicating substantial differences.

Abbreviations: CCI: Charlson Comorbidity Index; HCC: Hepatocellular Carcinoma; ICC: Intrahepatic Cholangiocarcinoma; PLC: Primary Liver Cancer; CI: confidence interval

Fig. S1: Patient flowchart

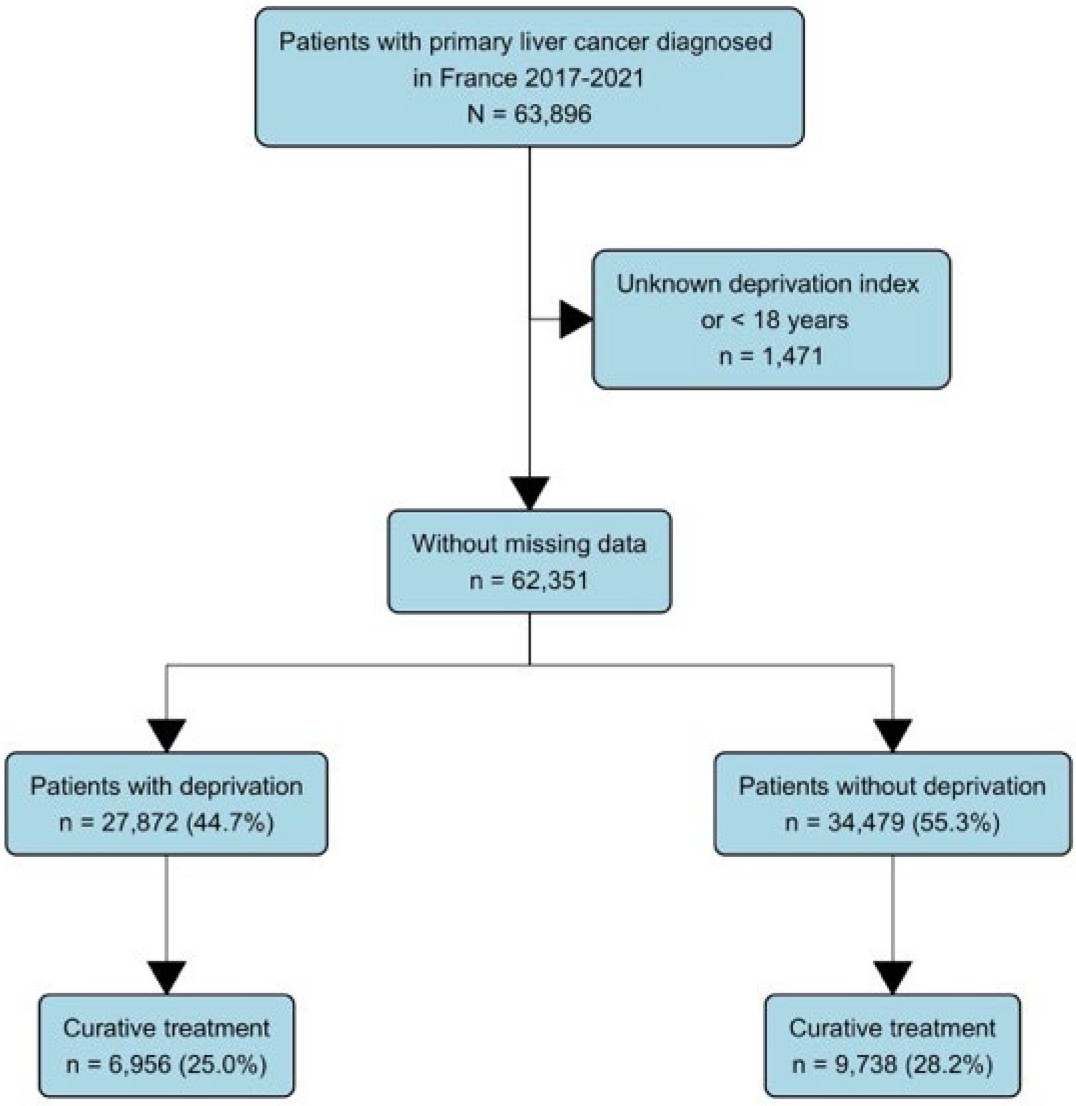

Fig. S2: Interactive map of the departmental variations of social deprivation and curative treatment access among patients with primary liver cancer in France January 1, 2017 - December 31, 2021

Variations of the 5-year incidence of social deprivation (A) and curative treatment access (B) among patients with primary liver cancer (PLC) along the 94 French regional departments between January 1, 2017 and December 31, 2021. Black dots represent expert hospitals in PLC management and dot diameter represents the number of PLC hospitalizations per hospital.

Available at: https://data.mendeley.com/preview/szbg4mnmxn?a=a5f6722b-f9f6-477a-a796-c5aed24f27df

Fig. S3: Correlation between 5-year incidence of deprivation and access to curative treatment in each of the 94 French regional departments.

Footnote: Each dot represents one French department

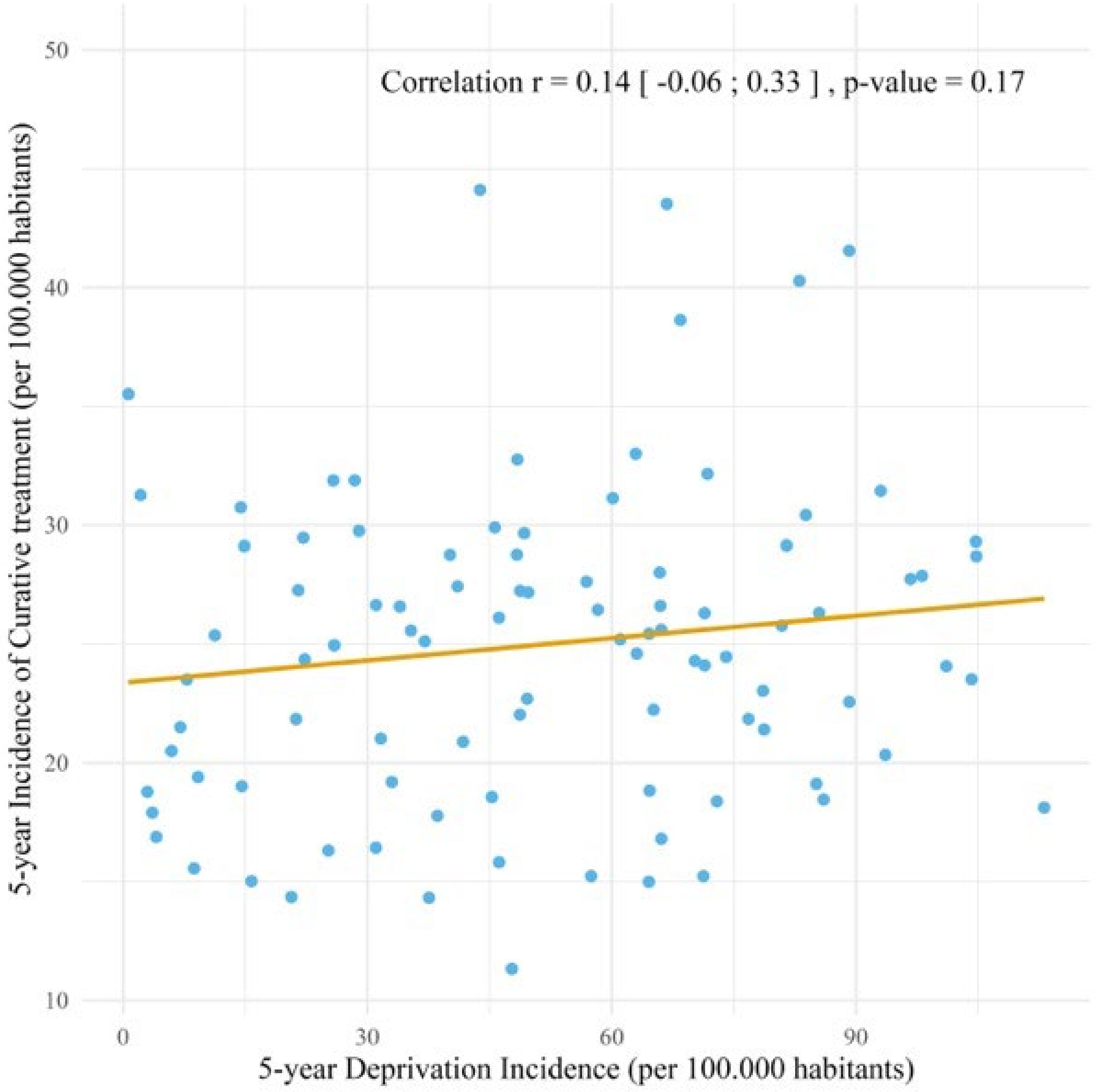

Fig. S4: Plot of confounding covariates before (red line) and after (blue line) balancing on a propensity score.

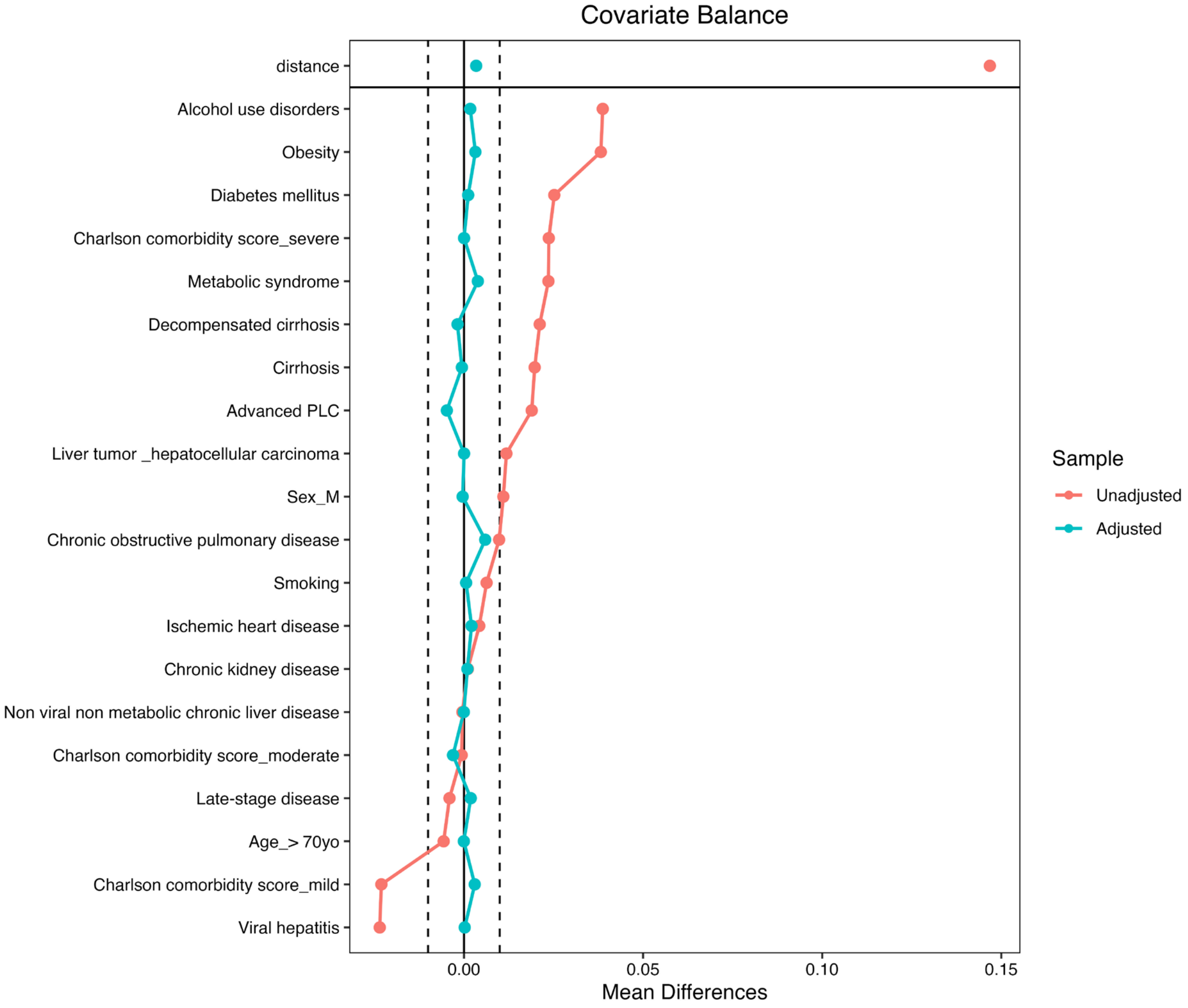

Footnote: Unbalanced general and liver-related comorbidities were included in the propensity score estimation using 1:1 nearest neighbor matching, caliper adjusted at 0.03 without replacement. Standardized mean differences (SMDs) were then used to assess covariate balance, with an SMD ≤ 0.01 (dotted line) indicating negligible differences, values from 0.0101 to 0.030 indicating very small differences, values from 0.0301 to 0.050 indicating small differences, and values > 0.050 indicating substantial differences.
